# Detection of Triacetone Triperoxide by High Kinetic Energy Ion Mobility Spectrometry

**DOI:** 10.1021/acs.analchem.3c04101

**Published:** 2023-11-10

**Authors:** Christoph Schaefer, Martin Lippmann, Michiel Beukers, Niels Beijer, Ben van de Kamp, Jaap Knotter, Stefan Zimmermann

**Affiliations:** † Institute of Electrical Engineering and Measurement Technology, Department of Sensors and Measurement Technology, Leibniz University Hannover, Appelstr. 9A, Hannover 30167, Germany; ‡ Research Group Technologies for Criminal Investigations, Saxion University of Applied Sciences, M.H Tromplaan 28, Enschede 7513AB, The Netherlands; § Knowledge Centre of Digitalization, Intelligence, and Technology, Police Academy of The Netherlands, Arnhemseweg 348, Apeldoorn 7334AC, The Netherlands

## Abstract

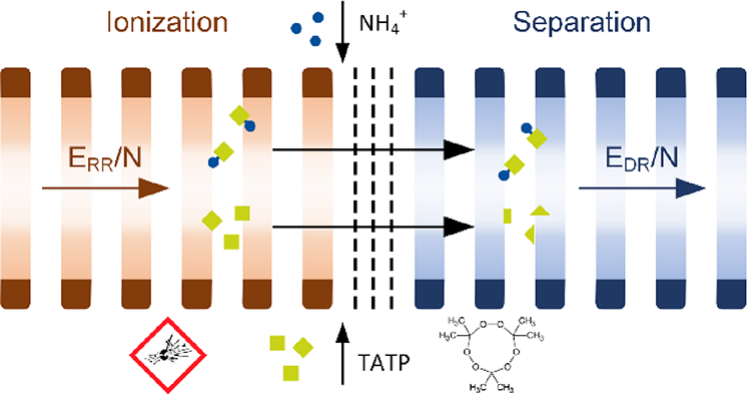

High Kinetic Energy Ion Mobility Spectrometry (HiKE-IMS) is a versatile technique for the detection of gaseous target molecules that is particularly useful in complex chemical environments, while the instrumental effort is low. Operating HiKE-IMS at reduced pressures from 10 to 60 mbar results in fewer ion-neutral collisions than at ambient pressure, reducing chemical cross-sensitivities and eliminating the need for a preceding separation dimension, e.g., by gas chromatography. In addition, HiKE-IMS allows operation over a wide range of reduced electric field strengths *E*/*N* up to 120 Td, allowing separation of ions by low-field ion mobility and exploiting the field dependence of ion mobility, potentially allowing separation of ion species at high *E*/*N* despite similar low-field ion mobilities. Given these advantages, HiKE-IMS can be a useful tool for trace gas analysis such as triacetone triperoxide (TATP) detection. In this study, we employed HiKE-IMS to detect TATP. We explore the ionization of TATP and the field-dependent ion mobilities, providing a database of the ion mobilities depending on *E*/*N*. Confirming the literature results, ionization of TATP by proton transfer with H_3_O^+^ in HiKE-IMS generates fragments, but using NH_4_^+^ as the primary reactant ion leads to the TATP·NH_4_^+^ adduct. This adduct fragments at high *E*/*N*, which could provide additional information for reliable detection of TATP. Thus, operating HiKE-IMS at variable *E*/*N* in the drift region generates a unique fingerprint of TATP made of all ion species related to TATP and their ion mobilities depending on *E*/*N*, potentially reducing the rate of false positives.

## Introduction

Triacetone triperoxide (TATP) is an unstable peroxide that is sensitive to heat, impact, and friction and exhibits an explosive power almost as strong as that of trinitrotoluene (TNT).^1^ Because its precursors are widely accessible to the general public and its synthesis is fast and simple without requiring special chemical skills, TATP poses danger to the public. As such, it has been used by terrorists as an explosive in the past.^2,3^ Moreover, TATP appears as a white powder, potentially making it difficult for police forces to differentiate it from other substances such as certain drugs of abuse. Therefore, a rapid and sensitive method to reliably detect TATP is necessary.

Ion mobility spectrometry (IMS) has been used to detect trace amounts of explosives since the 1970s^4,5^ due to its extreme sensitivity with low limits of detection in the single-digit ppt_v_ range in short measurement times down to 1 s.^6^ The possibility to build miniaturized IMS devices also renders them suitable for use as mobile and even hand-held instruments for field applications.^7−9^

Typically, IMS detects nitro-based explosives such as TNT, nitroglycerine (NG), and cyclotrimethylenetrinitramine (RDX) in negative polarity because of their high electron affinity and thus formation of negative product ions.^4,10^ The explosive TATP is an exception, as it is typically found in positive polarity.^11,12^

In complex chemical environments, especially when operating IMS at ambient pressure, analytes with higher proton affinity can remove the proton from analytes that are already ionized.^13−15^ As a result, substances with low proton affinity might not be detectable in IMS. To overcome this limitation, IMS is often coupled with gas chromatography (GC) to add a preceding separation dimension before sample introduction into the IMS.^16−19^ Ideally, the substances elute sequentially from the GC, allowing for individual ionization in the IMS without the presence of other substances with higher proton affinity to avoid proton removal of the newly formed ions. While this approach can reduce the effect of chemical cross-sensitivities, it also increases the measurement times to up to several minutes.^17,20,21^

Another method to mitigate cross-sensitivities caused by competing gas-phase reactions is to decrease the operating pressure of the instrument, leading to a lower number of collisions between ions and neutral molecules, shifting the ion population from thermodynamic equilibrium to kinetic control. This approach is used in High Kinetic Energy Ion Mobility Spectrometry (HiKE-IMS), operated at reduced pressures from 10 to 60 mbar.^22,23^ In addition to the mitigation of chemical cross-sensitivities,^24^ the operation at low pressure also allows to vary the reduced electric field strength *E*/*N* in a wide range up to 120 Td in both the reaction region and the drift region. Unlike drift tube IMS for trace gas detection operated at ambient pressure, HiKE-IMS separates ions not only by their low-field ion mobility but also by utilizing the field dependence of the ion mobility.^25−28^ Therefore, HiKE-IMS can help to separate ions despite similar low-field mobilities, thereby reducing false positives.^13^ Moreover, the operation at high *E*/*N* can lead to fragmentation of the formed product ions due to ion heating as the kinetic energy of the ions increases.^29−33^ Together with the signal from the parent ions at low *E*/*N*, a unique fragmentation pattern at high *E*/*N* can provide additional information about the analytes. In addition to these benefits, the lower number of ion-neutral collisions at the operating pressure of HiKE-IMS also leads to a decrease in sensitivity compared to IMS operated at ambient pressure for the detection of substances that are effectively ionized by atmospheric pressure chemical ionization at ambient pressure, especially under dry conditions.^13^ However, HiKE-IMS can show increased sensitivity for substances that are not effectively ionized by atmospheric pressure chemical ionization at ambient pressure. Furthermore, HiKE-IMS shows almost constant signal intensities of analytes that are difficult to ionize under humid conditions due to cluster dissociation of the reactant ions H_3_O^+^(H_2_O)_*n*_ at high *E*/*N* even when increasing background water concentrations up to 32,000 ppm_V_.^29^

Given these fundamental benefits, HiKE-IMS might also be a suitable tool for the detection of other trace gases. This study aims to demonstrate the utility of HiKE-IMS for detecting TATP as an exemplary application by investigating both product ion formation and field-dependent ion mobilities. In addition to the experiments with standalone HiKE-IMS, the ion species formed by TATP in HiKE-IMS have been identified by HiKE-IMS-MS to gain a better understanding of the behavior of TATP inside HiKE-IMS. Note, however, that for future field applications, the HiKE-IMS is supposed to be operated as a standalone instrument.

## Ion Formation of TATP

The ionization of TATP in various ion sources coupled to ion mobility spectrometry and mass spectrometry (MS), including atmospheric pressure chemical ionization (APCI), electrospray ionization (ESI), laser photoionization (LPI), and electron ionization (EI), has been studied in detail in the literature.^11,12,34−39^

Table 1 lists the ion species and their proposed molecular formulas found in the literature. Experiments in IMS operated at ambient pressure^11^ as well as in proton transfer reaction-MS (PTR-MS)^37^ and selected ion flow tube-MS (SIFT-MS)^36^ have demonstrated that fragments dominate the product ion population of TATP instead of the protonated TATP monomer. Ewing et al.^11^ did not detect the protonated TATP monomer in IMS, but it was found as a minor product ion in PTR-MS and SIFT-MS.^36,37^ However, both Ewing et al.^11^ and Tomlinson-Phillips et al.^12^ found a stable adduct between TATP and NH_4_^+^ with *m*/*z* 240 in IMS appearing in addition to fragments, by using ammonia as a dopant in the ion source generating NH_4_^+^(H_2_O)_*n*_ reactant ions. Likewise, when doping the ion source of PTR-MS with ammonia vapor, the same adduct between TATP and NH_4_^+^ is formed.^37^

**Table 1:** Ion Species Formed by TATP in Various Ion Sources for Both IMS and MS Have Been Reported in the Literature

molecular formula	*m*/*z*	instrument
C_2_H_3_O^+^	43	IMS-MS,^12^ PTR-MS,^37^ SIFT-MS,^36^ GC-MS (CI and EI),^38^ LPI-MS^35^
CH_3_CNH_2_^+^	43	IMS-MS^12^
C_3_H_6_NHH^+^	58	IMS-MS,^11,12^ PTR-MS,^37^ LPI-MS^35^
C_3_H_6_OH^+^	59	IMS-MS,^11^ SIFT-MS,^36^ GC-MS (CI and EI),^38^ LPI-MS^35^
C_3_H_9_CNH_2_^+^	73	IMS-MS^12^
C_3_H_6_O_2_^+^	74	PTR-MS,^37^ LC-MS,^34^ GC-MS (CI)^38^
C_3_H_6_O_2_H^+^	75	PTR-MS,^37^ GC-MS (CI and EI),^38^ LPI-MS^35^
(CH_3_)_3_CO_2_^+^	89	IMS-MS,^12^ LC-MS^34^
C_3_H_6_O_3_H^+^	91	IMS-MS,^11^ PTR-MS,^37^ SIFT-MS,^36^ LC-MS,^34^ GC-MS (CI)^38^
C_6_H_12_O_3_·H^+^	133	SIFT-MS^36^
C_9_H_18_O_6_·H^+^	223	PTR-MS,^37^ SIFT-MS^36^
C_9_H_18_O_6_·NH_4_^+^	240	IMS-MS,^11,12^ PTR-MS^37^

In addition, Tomlinson-Phillips et al.^12^ proposed the fragmentation pathways leading to the fragments (CH_3_)_3_CO_2_^+^ (*m*/*z* 89), C_3_H_9_CNH_2_^+^ (*m*/*z* 73), C_3_H_6_NHH^+^ (*m*/*z* 58), C_2_H_3_O^+^, and (CH_3_)CNH_2_^+^ (both *m*/*z* 43) by investigating isotopically labeled TATP with IMS coupled with triple-quad MS and supplying ammonia to the ion source.^12^ Other experiments involving TATP in laser photoionization MS, GC-MS with both chemical ionization (CI) via methane reactant ions and electron ionization, and liquid chromatography-MS (LC-MS) have found the same dominant fragments.^34,35,38^ Sigman et al. found in MS^3^ experiments that C_3_H_6_O_3_H^+^ (*m*/*z* 91) further fragments into C_3_H_6_O_2_^+^ (*m*/*z* 74) and ultimately C_2_H_3_O^+^ (*m*/*z* 43), which is supported by experiments in PTR-MS by Shen et al. at variable *E*/*N*.^37,38^

## Experimental Section

Detailed information regarding the design and function of HiKE-IMS can be found in previous publications.^22,40^ The corona discharge ion source, the reaction region, and the drift region are made of printed circuit boards (PCBs) of the same dimensions as in previous work.^41^Table 2 summarizes the relevant operating parameters of the HiKE-IMS.

**Table 2:** Operating Parameters of HiKE-IMS and HiKE-IMS-MS

parameter	value	value
instrument	standalone HiKE-IMS	HiKE-IMS-MS
reaction region length	50 mm	105 mm
drift region length	150.5 mm	153 mm
corona voltage	1050 V	1050 V
reaction region voltage	500–2050 V	1000–4300 V
drift region voltage	1500–6200 V	1500–6200 V
reduced reaction field strength *E*_RR_/*N* and reduced drift field strength *E*_DR_/*N*	30–120 Td	30–120 Td
injection time	3 μs	3 μs
drift gas flow rate^a^	10 mL/min	24 mL/min
sample gas flow rate^a^	12 mL/min	15 mL/min
operating pressure	16.7 mbar	14.3 mbar
temperature HiKE-IMS *T*_IMS_	80 °C	30 °C
temperature sample inlet *T*_Inlet_	200 °C	30 °C

a Gas flow rates correspond to reference conditions 293 K and 1013.25 hPa.

Figure S1 shows a schematic of the HiKE-IMS setup. Briefly, a corona discharge ion source is used to initiate the formation of reactant ions that subsequently ionize the neutral analyte molecules in the reaction region. A tristate ion shutter^40^ transfers ion packets into the drift region, where the different ion species are separated based on their ion mobility. The reduced reaction field strength *E*_RR_/*N* controlling the formation of reactant ions and the ionization of analyte molecules and the reduced drift field strength *E*_DR_/*N* affecting the separation of ions can be adjusted independently. Once separated in the drift region, the ions are collected by a Faraday plate, and the ion current is amplified using an in-house built transimpedance amplifier.^42^ The analog signal from the amplifier is converted into a digital signal using an analog-digital converter (ADQ14DC-2A-VG, Teledyne SP Devices, Sweden) with a sampling rate of 500 MSa/s.

A zero-air generator (ZA RACK FID 1.5, LNI Swissgas, Germany) in series with a combined moisture trap and hydrocarbon filter (ZPure Glass H_2_O/HC Filter, Chromatography Research Supplies, USA) supplies purified air used as the drift gas. The drift gas is supplied at the end of the drift region in the vicinity of the detector using a mass flow controller (F-200DV-ABD, Bronkhorst Nord, Germany) with a fixed flow rate of 10 mL/min (all given gas flow rates are referenced to 293 K and 1013.25 hPa). Figure S2 shows a schematic of the gas mixing system providing the sample gas. The sample gas is introduced into HiKE-IMS at the end of the reaction region near the tristate ion shutter. TATP was synthesized at the Knowledge Centre of Digitalization, Intelligence, and Technology of the Police Academy of The Netherlands. The sample is placed as powder in a closed container traversed by a fixed flow of nitrogen of 250 mL/min. A T-piece is positioned at the outlet of the container, where the HiKE-IMS draws sample gas at a flow rate of 12 mL/min through an inlet capillary. Hence, the headspace of the TATP sample is analyzed by HiKE-IMS. The flow rate of the sample gas is determined by the pressure difference between the instrument and the sample container and the geometry of the inlet capillary. With a fixed pressure difference, the flow rate is adjusted by the length of the capillary. The HiKE-IMS is evacuated near the corona needle using a membrane vacuum pump (MVP-003, Pfeiffer Vacuum, Germany), and the pressure is monitored by a capacitive pressure gauge (CMR 362, Pfeiffer Vacuum, Germany).

The experiments in this study are initially conducted without adding any dopant to the corona discharge ion source. In this so-called ambient ionization mode, the reactant ions H_3_O^+^, O_2_^+^, and NO^+^ dominate the reactant ion population. Subsequently, the experiments are conducted in the ammonia-doped ionization mode, in which a vacuum-tight container with a solution of ammonium chloride in water is connected to the interface between the corona discharge ion source and the reaction region via a capillary, see Figure S1, to shift the reactant ion population to NH_4_^+^.

To avoid sample condensation in the HiKE-IMS, thus ensuring rapid response and preventing prolonged contamination, the HiKE-IMS is heated up to 80 °C by placing the HiKE-IMS in a thermally insulated housing made of polyoxymethylene (POM). The internal temperature is regulated using custom-made heaters made of PCBs with conductor paths being meandered across to set the desired resistance depending on path geometry and specific resistance. By converting electrical power in the resistor, the PCBs are heated and individually regulated to the desired temperature using six individual temperature controllers (UR32742A, Wachendorff, Germany). The temperature is monitored at the detector of the HiKE-IMS using a Pt-1000 resistive temperature sensor (NB-PTCO-193, TE Connectivity, Germany). To prevent sample condensation in the sample inlet of the HiKE-IMS, the sample capillary is inserted into a heated sample inlet tube whose temperature can be increased up to 200 °C (KA2506PT4020-9, Mohr & Co, Germany). In Section S5, the temporal system response to TATP exposure at different operating temperatures is investigated; therefore, a second gas mixing system according to Figure S2 is used. This second gas mixing system incorporates a three-way valve to switch between TATP-enriched nitrogen and clean nitrogen.

The ions generated in HiKE-IMS are identified using HiKE-IMS-MS. More information on this instrument can be found in the literature,^29,43^ with the most relevant operating parameters listed in Table 2. The instrument was operated in the 2D-IMS-MS mode. In this mode, both ion gates, the one in front of the drift region and the one after the drift region, are opened for 3 μs with a fixed delay time. As a result, only a small selected mobility range of the ion mobility spectrum is transferred to the MS. By stepwise varying the delay time between the ion gate pulses and recording a mass spectrum for each delay time, a larger mobility range can be analyzed, thus reconstructing the ion mobility spectrum from the intensities of the individual *m*/*z* depending on delay time. In this work, the HiKE-IMS-MS is just used to identify the formed ion species and thus to gain an understanding of the behavior of TATP in HiKE-IMS to correctly interpret the results of a standalone HiKE-IMS, as required for future field applications. Hence, the measurements in this work are conducted using two different instruments: a standalone HiKE-IMS to record ion mobility spectra and determine reduced ion mobilities and a HiKE-IMS-MS to identify the generated ion species and help in understanding the ion formation of TATP in HiKE-IMS.

## Results and Discussion

The first experiments are conducted in the ambient ionization mode, where no dopant gas is supplied to the corona discharge ion source. Correspondingly, analyte ionization is expected via the reaction of H_3_O^+^, O_2_^+^, and NO^+^. To exploit the ability of HiKE-IMS to separate ions based on both their ion mobility and the field dependence of their ion mobility, a stepwise variation of the reduced drift field strength from 30 to 120 Td in increments of 1 Td was conducted at a constant reduced reaction field strength. The measurement was recorded both at a low *E*_RR_/*N* of 30 Td and at a high *E*_RR_/*N* of 100 Td. All reduced ion mobilities depending on *E*_DR_/*N* can be found in Tables S1 and S2 in the Supporting Information. For comparison, all measurements are repeated as blank measurements without adding TATP as shown in Figure S3.

### Ambient Ionization Mode

According to the blank measurements, H_3_O^+^ is the most abundant reactant ion at both *E*_RR_/*N* = 30 Td and *E*_RR_/*N* = 100 Td. Possibly, the moisture trap located downstream of the zero-air generator, which supplies air with a dew point of −20 °C, does not decrease the water concentration sufficiently for O_2_^+^ to have a significant abundance in the ion mobility spectrum. Unfortunately, no humidity sensors were available to measure the dew point of air leaving the moisture trap. Nevertheless, this benefits the current study, as the prevalence of H_3_O^+^ ensures that proton transfer is the major reaction pathway. Otherwise, significant contributions of O_2_^+^ to product ion formation complicate the interpretation of the ion mobility spectra due to the presence of additional ion species.

**Figure 1:**
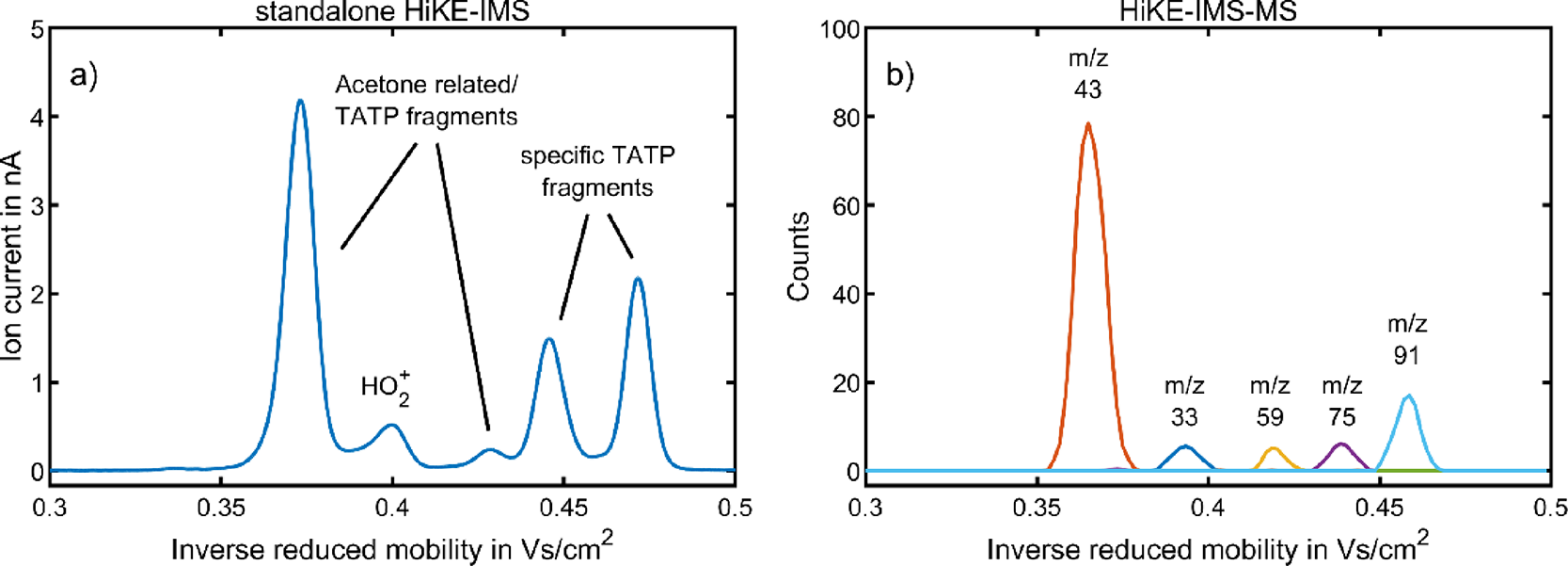
TATP with HiKE-IMS in the ambient ionization mode at *E*_RR_/*N* = *E*_DR_/*N* = 100 Td in air. (a) Positive ion mobility spectrum of TATP obtained with standalone HiKE-IMS. (b) Reconstructed ion mobility spectrum of TATP obtained with HiKE-IMS-MS operated in the 2D-IMS-MS mode showing the arrival time distributions of all ion species. All other operating parameters were set according to Table 2.

Figure 1 shows an exemplary ion mobility spectrum of TATP in HiKE-IMS as well as the arrival time distribution of all ion species measured by HiKE-IMS-MS at *E*_DR_/*N* = *E*_RR_/*N* = 100 Td. Two of the occurring ion species with *K*_0,1_ = 2.33 cm^2^/(V s) and *K*_0,2_ = 2.68 cm^2^/(V s), with mass-to-charge ratios of (*m*/*z*)_1_ 59 and (*m*/*z*)_2_ 43, respectively, are possibly the protonated monomer of acetone and the fragment C_2_H_3_O^+^ both of which have been found in HiKE-IMS-MS in a previous study on acetone.^29^ Confirming the previous results, Figure S5 shows that both ion species can also be found when introducing acetone to the standalone HiKE-IMS. Moreover, two additional peaks with mobilities of *K*_0,3_ = 2.24 cm^2^/(V s) and *K*_0,4_ = 2.12 cm^2^/(V s) and mass-to-charge ratios of (*m*/*z*)_3_ 75 and (*m*/*z*)_4_ 91, respectively, are found. As mentioned earlier, both of these fragments have been found for TATP in the literature, with proposed molecular formulas of C_3_H_6_O_2_H^+^ (*m*/*z* 75)^12^ and C_3_H_6_O_3_H^+^ (*m*/*z* 91).^11,36,37^ Furthermore, the reduced ion mobility of *K*_0,4_ = 2.12 cm^2^/(V s) for (*m*/*z*)_4_ 91 agrees well with the literature value of *K*_0_ = 2.14 cm^2^/(V s) obtained at a temperature of 120 °C.^11^ Note that Ewing et al. have demonstrated that the reduced ion mobility of *m*/*z* 91 increases from 2.00 to 2.20 cm^2^/(V s) when increasing the operating temperature from 60 to 180 °C. The ion species in the ion mobility spectrum with a reduced ion mobility of *K*_0,5_ = 2.5 cm^2^/(V s) and a mass-to-charge ratio of (*m*/*z*)_5_ 33 is likely originating from hydrogen peroxide residues from synthesis being ionized via hydrogen abstraction resulting in HO_2_^+^.

The exact origin of the ion species with *m*/*z* 43 and 59 is unclear. As acetone is a precursor in the synthesis of TATP, its presence in the headspace of TATP powder would not seem surprising. However, Sigman et al. also proposed that both of these ions can be formed as fragments from protonated TATP.^38^ Moreover, acetone might be formed as a dissociation product of TATP.^44^ However, the instrument cannot distinguish between ion formation by ionization of acetone in the reaction region and ionization of TATP and subsequent fragmentation.

In the ion mobility spectrum recorded with the standalone HiKE-IMS, no other ion species could be found outside the shown ion mobility range. Similarly, no other ion species could be found with HiKE-IMS-MS. Hence, other species related to TATP such as the dimer diacetone diperoxide (DADP) could not be detected in HiKE-IMS.

**Figure 2:**
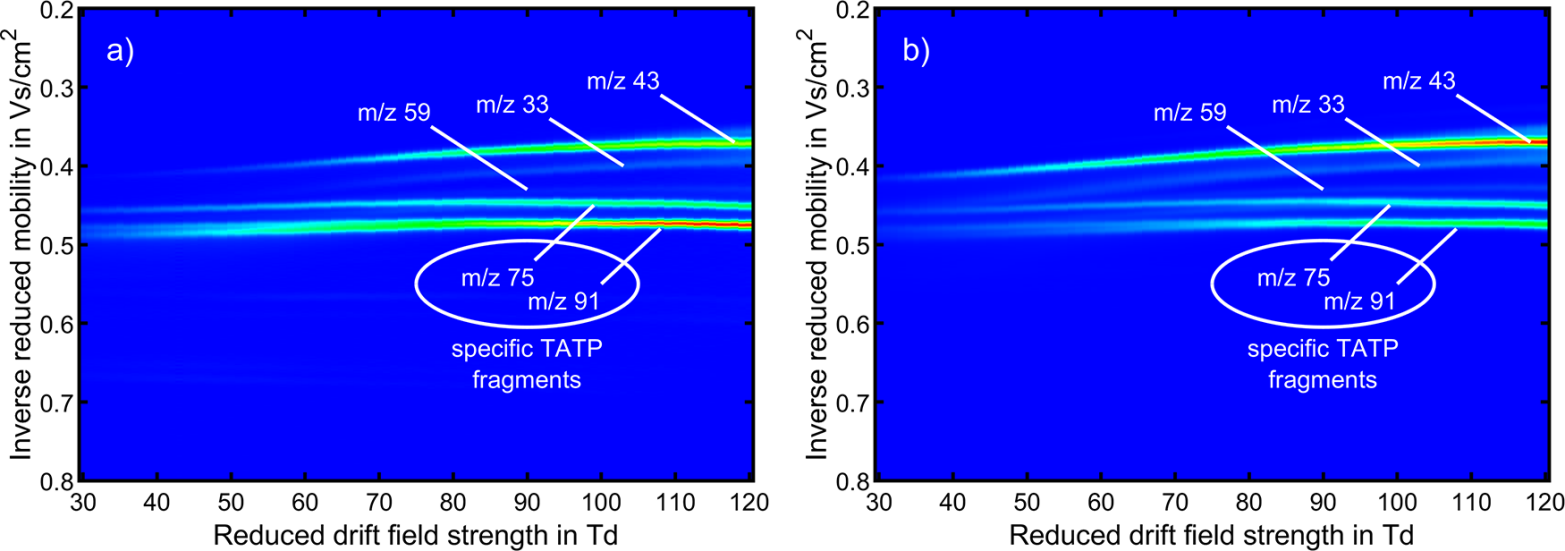
Dispersion plots of TATP recorded with standalone HiKE-IMS in the ambient ionization mode at *E*_RR_/*N* of (a) 30 and (b) 100 Td depending on *E*_DR_/*N* in air. The data are normalized to the maximum value of intensities of all spectra. The color denotes the intensity relative to the maximum value (red). All other operating parameters were set according to Table 2.

Figure 2 shows the results obtained from varying *E*_DR_/*N*. The individual ion mobility spectra recorded at different *E*_DR_/*N* values are combined into a single dispersion plot, visualizing the field dependence of the ion mobilities of all ion species. These data can serve as a database for the reduced ion mobility of TATP-related ion species in air depending on *E*_DR_/*N*. The results demonstrate that the ion mobility of all TATP-related ion species depends on the reduced drift field strength. However, the extent of the influence varies among the different ion species. For example, when increasing *E*_DR_/*N* from 30 to 120 Td, the reduced ion mobility of C_2_H_3_O^+^ (*m*/*z* 43) increases from 2.44 to 2.76 cm^2^/(V s). In contrast, the change in ion mobility between 30 and 100 Td for the ion species C_3_H_6_O_2_H^+^ (*m*/*z* 75) and C_3_H_6_O_3_H^+^ (*m*/*z* 91) is less pronounced as they increase from 2.22 to 2.28 cm^2^/(V s) for *m*/*z* 75 and from 2.11 to 2.16 cm^2^/(V s) for *m*/*z* 91. Upon further increasing *E*_DR_/*N*, both ion mobilities slightly decrease. The dispersion plot clearly shows that the signals for *m*/*z* 33 and 75 have similar ion mobilities below *E*_DR_/*N* = 40 Td. This highlights one major benefit of measuring at different *E*_DR_/*N* values using HiKE-IMS: Although the two ion species underlying the same peak in the ion mobility spectrum cannot be distinguished at low *E*_DR_/*N*, their separation is enabled at medium to high *E*_DR_/*N* by the differences in the field dependence of ion mobility. Therefore, measuring at different *E*_DR_/*N* values reveals more information than operation at a single *E*_DR_/*N*. Note that this phenomenon is expected to be more significant for ion species with reduced ion mobility strongly affected by *E*_DR_/*N*. Thus, varying *E*_DR_/*N* can help minimize false positive alarms caused by spectral interferences, i.e., ion species with similar ion mobility at low *E*_DR_/*N*, since it is more unlikely that the interfering ion species have the same field dependence of ion mobility.

Comparing the dispersion plots recorded at *E*_RR_/*N* = 30 Td and *E*_RR_/*N* = 100 Td shows that the fragments of TATP have different intensities depending on the *E*_RR_/*N*. While C_3_H_6_O_3_H^+^ (*m*/*z* 91) is the most abundant ion species at *E*_RR_/*N* = 30 Td, C_2_H_3_O^+^ (*m*/*z* 43) dominates the ion mobility spectrum at *E*_RR_/*N* = 100 Td. To investigate the influence of *E*_RR_/*N* on the formation of TATP-related fragments in more detail, a stepwise variation of *E*_RR_/*N* between 30 to 100 Td in increments of 1 Td in HiKE-IMS is conducted at a constant reduced drift field strength of *E*_DR_/*N* = 80 Td.

**Figure 3:**
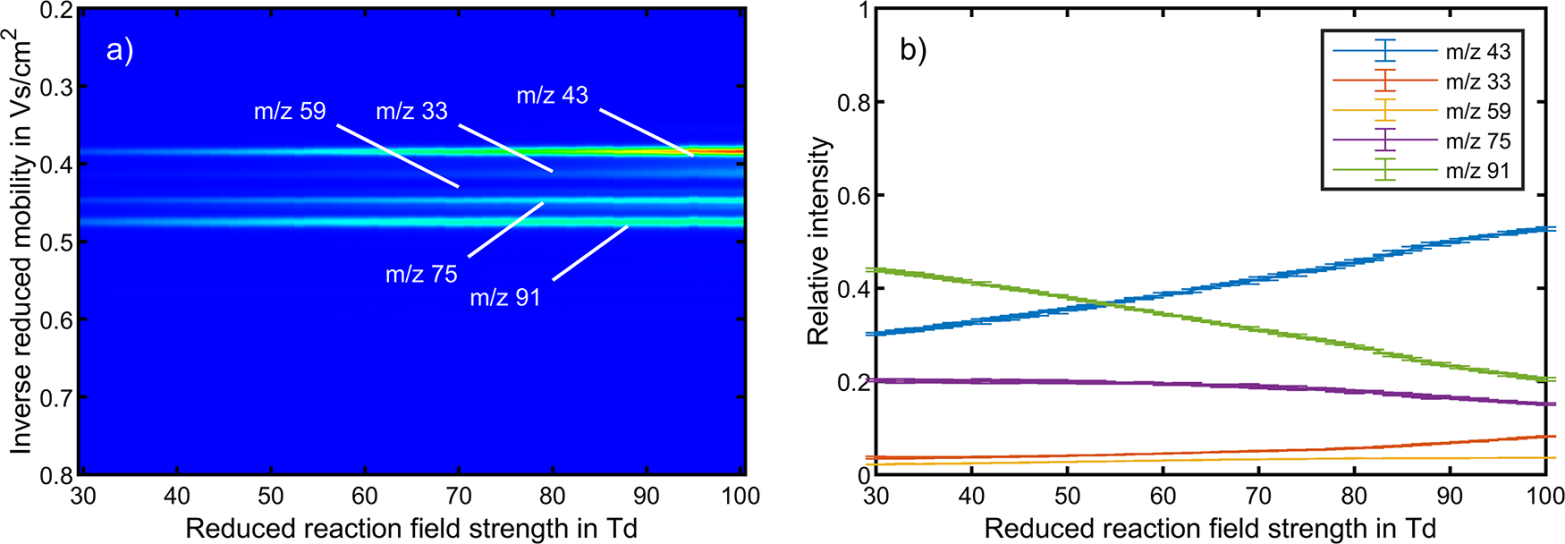
Investigation of the fragmentation of TATP-related product ions with standalone HiKE-IMS in the ambient ionization mode. (a) Reduced ion mobilities of TATP in air depending on *E*_RR_/*N* in the ambient ionization mode at *E*_DR_/*N* = 80 Td. The data are normalized to the maximum value of intensities of all spectra. The color denotes the intensity relative to the maximum value (red). (b) Relative intensities of the individual ion species extracted from the ion mobility spectra recorded with HiKE-IMS. Relative intensities are determined by normalizing the peak area of the specific product ion to the total integral of all formed ions. All other operating parameters were set according to Table 2.

The reduced ion mobilities of TATP-related peaks in Figure 3a are not really affected by changing *E*_RR_/*N*. This is expected since only *E*_DR_/*N* should affect the separation of the ion species in the drift region. Previous work has shown that varying the *E*_RR_/*N* has only a small influence on the ion mobilities in HiKE-IMS.^25^ A difference is observed only in cases where the cluster size of ions in the reaction region differs from that in the drift region and the time to establish a new equilibrium in the drift region is long enough to significantly impact drift times.

Although *E*_RR_/*N* has a negligible impact on ion mobilities, it has a more significant influence on the signal intensities of the fragments. At a low *E*_RR_/*N* of 30 Td, C_2_H_3_O^+^ (*m*/*z* 43) constitutes about 30% of the product ions, whereas C_3_H_6_O_3_H^+^ (*m*/*z* 91) accounts for about 44%. However, as *E*_RR_/*N* increases, the relative abundance of *m*/*z* 43 increases to 52% at *E*_RR_/*N* = 100 Td, whereas the relative abundance of *m*/*z* 91 decreases to 20%. Possibly, C_3_H_6_O_3_H^+^ (*m*/*z* 91) fragments due to the increase in ion energy at higher *E*_RR_/*N* forming C_2_H_3_O^+^ (*m*/*z* 43). This fragmentation pathway has already been proposed in experiments in PTR-MS and GC-MS in the literature.^37,38^ In contrast, when changing *E*_RR_/*N,* the relative abundance of the other ion species is less affected. When choosing a method for detecting TATP with HiKE-IMS, the fragmentation of C_3_H_6_O_3_H^+^ forming C_2_H_3_O^+^ should be taken into account since the same fragment C_2_H_3_O^+^ is also formed when introducing acetone or 2-butanone into HiKE-IMS and is thus not specific to TATP.^29^ For more specific detection of TATP, providing ammonia vapor to the ion source to shift the reactant ion population to NH_4_^+^ thus forming a stable and specific adduct TATP·NH_4_^+^ is suggested in the literature.^11,12,37,38^ To verify that the same adduct can be observed in HiKE-IMS, the above experiments are repeated in the ammonia-doped ionization mode.

### Ammonia-Doped Ionization Mode

To analyze the field dependence of ion mobility for the product ions in the ammonia-doped ionization mode, the same incremental variation of *E*_DR_/*N* from 30 to 120 Td in increments of 1 Td is conducted. Figure S4 shows the dispersion plots of blank measurements in the ammonia-doped ionization mode at *E*_RR_/*N* = 30 Td and *E*_RR_/*N* = 100 Td without adding TATP. Tables S3 and S4 include all determined reduced ion mobilities depending on *E*_DR_/*N*. In the ammonia-doped ionization mode at *E*_RR_/*N* = 30 Td, NH_4_^+^ is the most abundant ion species. Increasing *E*_RR_/*N* to 100 Td, the presence of reactant ions NO^+^, H_3_O^+^, and O_2_^+^ is confirmed by HiKE-IMS-MS; however, they are less abundant than NH_4_^+^. Thus, the product ion population is expected to be dominated by ions formed by reactions with NH_4_^+^.

**Figure 4:**
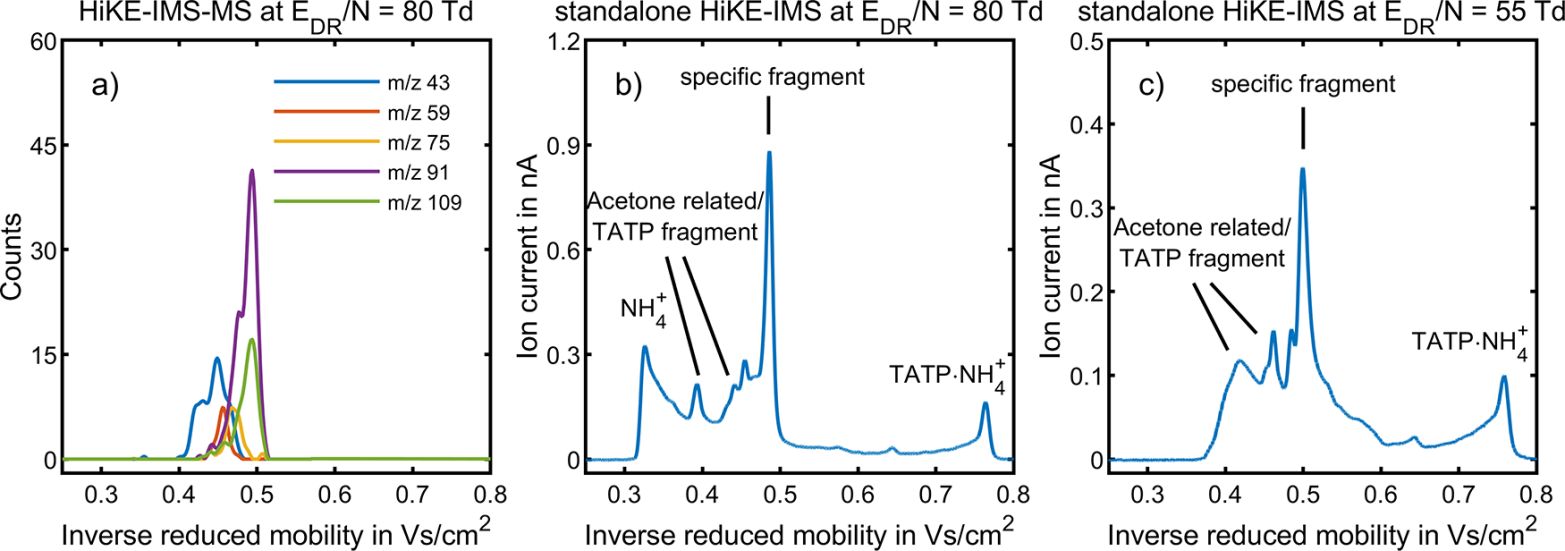
TATP with HiKE-IMS-MS and standalone HiKE-IMS in the ammonia-doped ionization mode at *E*_RR_/*N* = 30 Td in air. (a) Reconstructed ion mobility spectrum of TATP obtained with HiKE-IMS-MS operated in the 2D-IMS-MS mode showing the arrival time distribution for each ion species at *E*_DR_/*N* = 80 Td. (b) Positive ion mobility spectrum of TATP obtained with standalone HiKE-IMS at *E*_DR_/*N* = 80 Td. (c) Positive ion mobility spectrum of TATP obtained with standalone HiKE-IMS at *E*_DR_/*N* = 55 Td. All other operating parameters were set according to Table 2.

Figure 4 shows an exemplary ion mobility spectrum of TATP in the ammonia-doped ionization mode at *E*_DR_/*N* = 80 Td and *E*_RR_/*N* = 30 Td recorded with HiKE-IMS as well as the reconstructed ion mobility spectrum from HiKE-IMS-MS. Several additional peaks can be observed in the ion mobility spectrum when compared to the blank measurements without TATP. Although having slightly lower reduced ion mobilities than in the ambient ionization mode, the same fragments C_2_H_3_O^+^ (*m*/*z* 43), C_3_H_6_OH^+^ (*m*/*z* 59), C_3_H_6_O_2_H^+^ (*m*/*z* 75), and C_3_H_6_O_3_H^+^ (*m*/*z* 91) can be found in the ammonia-doped ionization mode, as identified by HiKE-IMS-MS. In addition, a hydrate C_3_H_6_O_3_H^+^·H_2_O (*m*/*z* 109) can be found as well with the same ion mobility as the fragment C_3_H_6_O_3_H^+^ since both ion species are in equilibrium in the drift region due to a fast association reaction with H_2_O and subsequent cluster dissociation. As can be seen, the HiKE-IMS-MS spectrum in Figure 4 a shows a broadening of the arrival time distributions of *m*/*z* 91 and *m*/*z* 43 indicating fragmentation in the drift region forming C_2_H_3_O^+^ from C_3_H_6_O_3_H^+^. The presence of multiple fragments in the mobility range between *K*_0_ = 2 and 2.5 cm^2^/(V s) (1/*K*_0_ = 0.4 to 0.5 Vs/cm^2^) explains the raised baseline in the ion mobility spectrum in Figure 4b. Since fragmentation is a statistical process occurring in the drift region, the ion mobility as determined by the instrument is a weighted average of the ion mobilities of the individual ion species as the ion drifts as weighted by the fraction of the drift region in which the ion drifts as either species. While the peak shape in the mobility range where the fragments occur is in good agreement in both HiKE-IMS and HiKE-IMS-MS operated at *E*_DR_/*N* = 80 Td, the HiKE-IMS-MS does not detect the ion species with the highest reduced ion mobility of *K*_0_ = 3.13 cm^2^/(V s) (1/*K*_0_ = 0.32 Vs/cm^2^), which is the most abundant ion species in the blank measurements and is thus probably the reactant ion NH_4_^+^, and the ion species with the lowest reduced ion mobility of *K*_0_ = 1.32 cm^2^/(V s) (1/*K*_0_ = 0.76 Vs/cm^2^). Since the ion mobility of *K*_0_ = 1.32 cm^2^/(V s) differs only slightly from the literature value for the reduced ion mobility of the adduct TATP·NH_4_^+^ of *K*_0_ = 1.36 cm^2^/(V s),^11^ the ion species found in HiKE-IMS might be the same adduct. Unfortunately, the adduct (*m*/*z* = 240) cannot be found in HiKE-IMS-MS even at low *E*/*N*, possibly due to fragmentation in the transfer region. Nevertheless, due to the good agreement of the determined reduced ion mobility with the literature value and since the adduct was also found in the literature when TATP was ionized with NH_4_^+^, we assume that the peak in HiKE-IMS is the adduct TATP·NH_4_^+^. The elevated baseline between the signal of the adduct TATP·NH_4_^+^ and the fragments suggests that in this experiment, the fragments and NH_4_^+^ are formed by fragmentation of the adduct in the drift region. Thus, the difference in operating temperature of the standalone HiKE-IMS of 80 °C and the HiKE-IMS-MS of 30 °C could explain the absence of the ion species NH_4_^+^ in HiKE-IMS-MS since it causes a difference in effective temperature and thus kinetic energy at the same *E*_DR_/*N*. Since the effective temperature depends on reduced ion mobility, especially the ions with lower reduced ion mobility such as the adduct TATP·NH_4_^+^ have a larger relative difference in effective temperature for the same difference in operating temperature. For example, the adduct TATP·NH_4_^+^ has an effective temperature of 448 K in the standalone HiKE-IMS and 398 K in HiKE-IMS-MS, both operated at *E*_DR_/*N* = 80 Td. Since the data indicate that the fragments are formed via fragmentation of TATP·NH_4_^+^ in the drift region, for this special case, the reconstructed ion mobility spectrum from HiKE-IMS-MS has to be compared to an ion mobility spectrum from the standalone HiKE-IMS at lower *E*_DR_/*N*, where the adduct has the same effective temperature of 398 K, corresponding to a reduced drift field strength of *E*_DR_/*N* = 55 Td. Comparing Figure 4a and c, it is apparent that neither HiKE-IMS-MS at *E*_DR_/*N* = 80 Td nor the standalone HiKE-IMS at *E*_DR_/*N* = 55 Td shows the ion species NH_4_^+^, possibly since the effective temperature of the adduct TATP·NH_4_^+^ is insufficient for the formation of NH_4_^+^. Also, the peak shapes in the inverse ion mobility range between 0.4 and 0.5 Vs/cm^2^, where the fragments occur, agree well between HiKE-IMS-MS at *E*_DR_/*N* = 80 Td and the standalone HiKE-IMS at *E*_DR_/*N* = 55 Td. The comparison of ion mobility spectra at different *E*_DR_/*N* values was not required in analysis of Figure 1 in the ambient ionization mode due to the smaller relative differences in effective temperature. Since the kinetic energy of the ions already appears to be sufficient for complete fragmentation of TATP in the reaction region, the difference in operating temperature may lead to a small difference in reduced ion mobility, which is, however, small enough for identification of the ion species in the standalone HiKE-IMS with the data from HiKE-IMS-MS.

**Figure 5:**
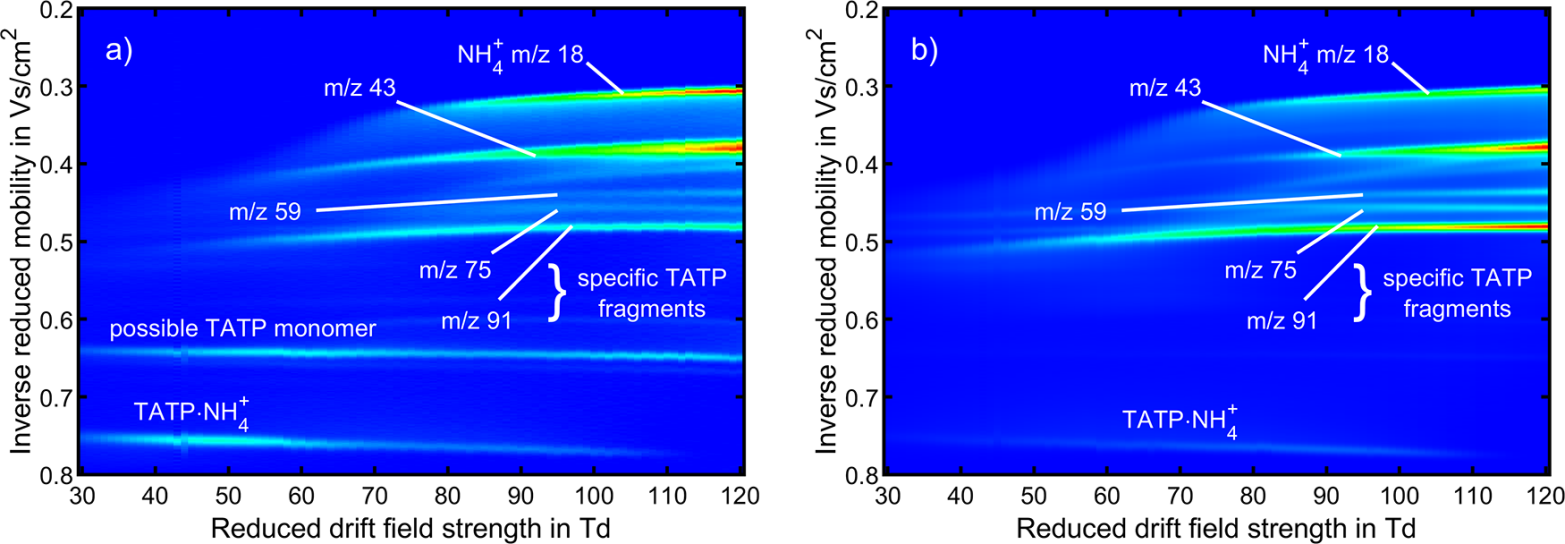
Dispersion plots of TATP recorded with standalone HiKE-IMS in the ammonia-doped ionization mode at *E*_RR_/*N* of (a) 30 and (b) 100 Td depending on *E*_DR_/*N* in air. The data are normalized to the maximum value of intensities of all spectra. The color denotes the intensity relative to the maximum value (red). All other operating parameters were set according to Table 2.

The dispersion plots of TATP in Figure 5 compared to those of the blank measurements in the ammonia-doped ionization mode shown in Figure S4 reveal six additional ion species that are formed when adding TATP. Five of these have already been identified as the same fragments that also occur in the ambient ionization mode and the adduct TATP·NH_4_^+^. The identity of the ion species with *K*_0_ = 1.56 cm^2^/(V s) at 30 Td is unclear since it was not found in HiKE-IMS-MS. Perhaps, this ion species is the protonated monomer, which was not detected in the ambient ionization mode. The softer ionization of TATP by adduct formation with NH_4_^+^ and subsequent cluster dissociation may result in the formation of a protonated monomer in HiKE-IMS. The ion species is detected only at *E*_RR_/*N* = 30 Td but not significantly at *E*_RR_/*N* = 100 Td, indicating fragmentation at higher *E*/*N*. While this behavior is consistent with the observations in IMS and MS, indicating that the protonated monomer of TATP is not stable, it is not possible to conclusively identify the protonated monomer based on these indications. The ion mobility spectrum at *E*_RR_/*N* = 30 Td and *E*_DR_/*N* = 50 Td in Figure S6 shows that both the adduct TATP·NH_4_^+^ and the possible protonated monomer form sharper peaks at low *E*_DR_/*N*, where the ions fragment less due to their lower kinetic energy.

The dispersion plots in Figure 5 highlight the significant influence of *E*_DR_/*N* on the reduced ion mobilities for most ion species. Especially at low *E*_DR_/*N*, the ion mobility of faster ion species with reduced ion mobilities above *K*_0_ = 2 cm^2^/(V s) increases significantly. Additionally, these ion species form distinct peaks in the ion mobility spectrum only at reduced drift field strengths above 60 to 80 Td. Moreover, the raised baseline between the peaks in the ion mobility spectrum indicates fragmentation in the drift region. Correspondingly, at low *E*_DR_/*N*, where the effective temperatures are insufficient for fragmentation, only the adduct TATP·NH_4_^+^ and the possible monomer are detected. Another explanation for the observed field dependencies of ion mobility is ion–solvent cluster formation. The ions may form clusters with NH_3_ at low *E*_DR_/*N*, leading to an increase in the collision cross section that is most significant in the case of the smaller and faster ions. When increasing *E*_DR_/*N*, collision-induced cluster dissociation leads to a decrease in the collision cross section, resulting in the shift toward higher reduced ion mobilities. In contrast, larger ions with lower reduced ion mobilities appear less prone to clustering at low *E*_DR_/*N*; thus, increasing the reduced drift field strength does not result in a significant increase in reduced ion mobilities.

Comparing the experiments conducted at *E*_RR_/*N* of 30 Td with *E*_RR_/*N* = 100 Td, it is apparent that the intensity of the adduct TATP·NH_4_^+^ decreases as *E*_RR_/*N* increases. Probably, the adduct forms fragments due to ion heating at high *E*_RR_/*N*. Shen et al. also observed a similar trend for the adduct above 100 Td in PTR-MS.^37^ Nevertheless, even at the highest reduced field strengths of *E*_RR_/*N* = 100 Td and *E*_DR_/*N* = 120 Td, the adduct TATP·NH_4_^+^ can still be detected with small intensities. Furthermore, when operating HiKE-IMS at 100 Td, C_3_H_6_O_3_H^+^ (*m*/*z* 91) has a relative abundance significantly higher than that at 30 Td. This could be attributed to the adduct dissociating to form the fragment with *m*/*z* 91.

In summary, the measurements in the ammonia-doped ionization mode show the presence of an adduct TATP·NH_4_^+^, especially at low *E*_RR_/*N*, which is more specific to detect TATP than the fragments present in the ambient ionization mode. Anyway, the dispersion plots recorded with the HiKE-IMS create a unique fingerprint of TATP made of all ion species related to TATP and their ion mobilities depending on *E*_DR_/*N*, especially when accompanied by the presence of fragments at high *E*_DR_/*N*, both of which can help to reduce false positives when detecting TATP. Note that the increment to be selected for *E*_DR_/*N* in practical applications depends on both the required amount of additional information and the available measurement time. One possible method that provides additional information for the detection of TATP in quasi-real time is to operate HiKE-IMS in the ammonia-doped ionization mode at comparably low *E*_DR_/*N* such as 50 Td, where the specific adduct has a sharp signal as shown in Figure 5 and in the ion mobility spectrum in Figure S6. In the case of an alarm, rapid switching to high *E*_DR_/*N* can then either confirm or disprove the alarm, depending on whether the fragments are present or not.

## Conclusions

In this work, we demonstrate the capability of HiKE-IMS for reliable detection of TATP based on the unique fingerprint of TATP made of all ion species, including fragments related to TATP and their ion mobilities depending on *E*/*N* as an exemplary application in trace gas analysis. In HiKE-IMS, TATP behaves similarly to experiments with IMS and MS reported in the literature. In the ambient ionization mode dominated by proton transfer reactions giving the product ions, only fragments of TATP are detected. However, with the HiKE-IMS operated in the ammonia-doped ionization mode at low *E*/*N*, a stable adduct TATP·NH_4_^+^ is formed. Thus, HiKE-IMS in the ammonia-doped ionization mode combines specific ionization through the formation of an adduct with NH_4_^+^ at low *E*/*N* with the formation of additional TATP-related fragments at high *E*/*N*, which can help to reduce false positives.

In addition, the dispersion plots of reduced ion mobilities depending on *E*_DR_/*N* reveal another benefit of using HiKE-IMS, i.e., the ability to simultaneously determine both the reduced ion mobility and the field dependence of ion mobility. Accordingly, HiKE-IMS can help to separate ion species with similar ion mobilities at a single *E*_DR_/*N* but different field dependencies of ion mobility. Varying *E*_DR_/*N* can create a unique fingerprint, further reducing false positives. Thus, we suggest the HiKE-IMS as a suitable instrument for reliably detecting TATP. One aim of this paper is to understand the behavior of TATP in HiKE-IMS by using a HiKE-IMS-MS instrument to provide a basis for future applications of field-ready standalone HiKE-IMS. Although this study focuses on TATP, HiKE-IMS can have benefits beyond TATP detection and can be useful in other applications, especially in complex chemical environments. Future work will evaluate HiKE-IMS for other applications, including the detection of drugs of abuse with a focus on the impact of interferences on trace gas analysis with HiKE-IMS.

## Supporting Information Available

Tables of the reduced ion mobilities of all ion species in the ambient ionization mode and in the ammonia-doped ionization mode, schematic of the HiKE-IMS, schematic of the gas mixing system, blank dispersion plots in the ambient ionization mode, blank dispersion plots in the ammonia-doped ionization mode, comparison of ion mobility spectra of a headspace measurement of both acetone and TATP, ion mobility spectrum of TATP in the ammonia-doped ionization mode at *E*_DR_/*N* = 50 Td, and system response to TATP (PDF)
