# Supplementary material for: Detection of Triacetone Triperoxide by High Kinetic Energy Ion Mobility Spectrometry
Source: Anal Chem. 2023 Nov 10;95(46):17099–107. doi: 10.1021/acs.analchem.3c04101 (PMC10666079; doi:10.1021/acs.analchem.3c04101)
Supplement: Supplementary file 1 — ac3c04101_si_001.pdf [file ac3c04101_si_001.pdf]

Supporting Information

Detection of Triacetone Triperoxide (TATP) by High Kinetic Energy Ion Mobility Spectrometry

Christoph Schaefer[1*], Martin Lippmann[1], Michiel Beukers[2,3], Niels Beijer[2,3], Ben van de Kamp[2,3], Jaap Knotter[2,3], Stefan Zimmermann[1]

[1] Leibniz University Hannover, Institute of Electrical Engineering and Measurement Technology, Department of Sensors and Measurement Technology, Appelstr. 9A, 30167 Hannover, Germany

[2] Research Group Technologies for Criminal Investigations, Saxion University of Applied Sciences, M.H Tromplaan 28, 7513AB, Enschede, The Netherlands

[3] Knowledge Centre of Digitalization, Intelligence and Technology, Police Academy of the Netherlands, Arnhemseweg 348, 7334AC Apeldoorn, The Netherlands

*Corresponding Author: schaefer@geml.uni-hannover.de

Table of contents

Table S1. Reduced ion mobilities of TATP-related product ions in ambient ionization mode in air depending on E_{DR}/N 2

Table S2. Reduced ion mobilities of reactant ions in ambient ionization mode in air depending on E_{DR}/N 6

Table S3. Reduced ion mobilities of TATP-related product ions in ammonia-doped ionization mode in air depending on E_{DR}/N 10

Table S4. Reduced ion mobilities of reactant ions in ammonia-doped ionization mode in air depending on E_{DR}/N 14

Figure S1. Schematic of the HiKE-IMS 18

Figure S2. Schematic of the gas mixing system 19

Figure S3. Blank dispersion plot in ambient ionization mode without adding TATP depending on E_{DR}/N in air 20

Figure S4. Blank dispersion plot in ammonia-doped ionization mode without adding TATP depending on E_{DR}/N in air 20

Figure S5. Comparison between ion mobility spectra of a blank measurement, acetone, and TATP at $E_{DR}/N = E_{RR}/N$ = 100 Td in air 21

Figure S6. Positive ion mobility spectrum of TATP in ammonia-doped ionization mode at $E_{DR}/N = E_{RR}/N$ = 30 Td in air 22

Figure S7. System response to TATP at different operating temperatures 24

S1. Reduced Ion Mobilities

Table S1. Recorded reduced ion mobilities K_0 of the ions $C_2H_3O^+$ (m/z 43), $C_3H_6OH^+$ (m/z 59), $C_3H_6O_2H^+$ (m/z 75) and $C_3H_6O_3H^+$ (m/z 91) in ambient ionization mode in air depending on reduced drift field strength E_{DR}/N at E_{RR}/N = 30 Td and E_{RR}/N = 100 Td. The reduced ion mobilities of TATP-related product ions were determined from measurements where TATP was supplied using the gas mixing system in *Figure S2* a). All other operating parameters were set according to Table 2 in the main manuscript.

	$C_2H_3O^+$ (m/z 43)		$C_3H_6OH^+$ (m/z 59)		$C_3H_6O_2H^+$ (m/z 75)		$C_3H_6O_3H^+$ (m/z 91)	
E_{RR}/N in Td	30 Td	100 Td	30 Td	100 Td	30 Td	100 Td	30 Td	100 Td
E_{DR}/N in Td	K_0 in cm^2/Vs	K_0 in cm^2/Vs	K_0 in cm^2/Vs	K_0 in cm^2/Vs	K_0 in cm^2/Vs	K_0 in cm^2/Vs	K_0 in cm^2/Vs	K_0 in cm^2/Vs
30		2.443			2.232	2.222	2.102	2.111
31		2.448			2.232	2.225	2.102	2.113
32		2.449			2.234	2.225	2.104	2.113
33		2.450			2.231	2.224	2.100	2.111
34		2.456			2.234	2.228	2.104	2.115
35		2.454			2.236	2.225	2.106	2.111
36		2.458			2.236	2.226	2.105	2.113
37		2.466			2.236	2.232	2.106	2.117
38		2.466			2.239	2.230	2.108	2.116
39		2.471			2.239	2.232	2.108	2.117
40		2.472			2.241	2.232	2.109	2.116
41		2.481			2.241	2.237	2.109	2.122
42	2.490	2.488			2.241	2.241	2.109	2.125
43	2.495	2.490			2.243	2.241	2.111	2.125
44	2.497	2.492			2.243	2.240	2.111	2.124
45	2.501	2.495			2.245	2.239	2.112	2.124
46	2.504	2.498			2.246	2.237	2.113	2.120
47	2.508	2.500			2.247	2.245	2.114	2.120
48	2.511	2.507			2.248	2.238	2.115	2.126

49	2.514	2.510			2.248	2.237	2.115	2.126
50	2.522	2.518			2.253	2.243	2.119	2.130
51	2.522	2.521			2.251	2.243	2.117	2.130
52	2.529	2.525			2.255	2.245	2.120	2.130
53	2.532	2.530			2.255	2.248	2.120	2.130
54	2.539	2.532			2.259	2.247	2.123	2.128
55	2.542	2.539			2.259	2.251	2.124	2.130
56	2.546	2.548			2.260	2.257	2.124	2.133
57	2.550	2.551			2.261	2.257	2.125	2.132
58	2.554	2.559			2.262	2.261	2.126	2.135
59	2.560	2.558			2.265	2.259	2.128	2.131
60	2.563	2.558			2.265	2.256	2.128	2.128
61	2.570	2.564			2.268	2.259	2.132	2.130
62	2.573	2.573			2.268	2.263	2.132	2.134
63	2.578	2.579			2.269	2.266	2.133	2.136
64	2.584	2.585			2.272	2.268	2.136	2.138
65	2.587	2.592			2.271	2.271	2.135	2.128
66	2.593	2.595			2.273	2.271	2.138	2.130
67	2.597	2.598			2.274	2.271	2.139	2.130
68	2.601	2.602			2.274	2.271	2.139	2.132
69	2.604	2.608			2.274	2.273	2.139	2.135
70	2.611	2.607			2.277	2.269	2.142	2.133
71	2.615	2.615			2.277	2.273	2.143	2.139
72	2.618	2.621			2.277	2.275	2.143	2.144
73	2.625	2.629			2.279	2.278	2.145	2.147
74	2.628	2.632			2.278	2.278	2.145	2.146
75	2.632	2.634			2.279	2.277	2.146	2.146
76	2.636	2.641			2.279	2.279	2.147	2.148

77	2.641	2.647			2.280	2.280	2.148	2.150
78	2.646	2.647			2.281	2.278	2.150	2.147
79	2.652	2.653			2.283	2.280	2.152	2.150
80	2.655	2.659			2.282	2.282	2.151	2.151
81	2.659	2.663			2.283	2.282	2.152	2.152
82	2.664	2.669			2.284	2.284	2.154	2.154
83	2.670	2.675		2.305	2.286	2.285	2.155	2.156
84	2.673	2.680		2.309	2.285	2.287	2.155	2.158
85	2.676	2.687		2.313	2.285	2.289	2.155	2.160
86	2.679	2.683		2.310	2.285	2.284	2.155	2.155
87	2.684	2.687		2.311	2.286	2.284	2.157	2.155
88	2.686	2.691		2.313	2.285	2.284	2.156	2.156
89	2.689	2.696		2.316	2.285	2.286	2.156	2.158
90	2.691	2.701		2.318	2.284	2.286	2.155	2.159
91	2.695	2.702		2.318	2.284	2.285	2.156	2.158
92	2.697	2.704		2.323	2.283	2.284	2.155	2.157
93	2.701	2.705		2.329	2.283	2.282	2.156	2.156
94	2.708	2.710		2.347	2.286	2.284	2.159	2.157
95	2.708	2.715		2.353	2.283	2.285	2.157	2.159
96	2.708	2.720		2.360	2.281	2.286	2.155	2.160
97	2.710	2.723		2.362	2.280	2.285	2.154	2.160
98	2.715	2.721		2.362	2.281	2.282	2.156	2.157
99	2.716	2.723		2.363	2.280	2.281	2.155	2.156
100	2.717	2.728		2.368	2.278	2.282	2.153	2.158
101	2.721	2.733		2.371	2.278	2.284	2.154	2.159
102	2.722	2.734		2.372	2.277	2.282	2.154	2.159
103	2.728	2.735		2.372	2.280	2.281	2.156	2.157
104	2.729	2.737		2.373	2.278	2.280	2.155	2.157

105	2.732	2.739	2.317	2.374	2.278	2.279	2.155	2.157
106	2.734	2.740	2.312	2.374	2.278	2.278	2.155	2.156
107	2.735	2.745	2.318	2.378	2.276	2.279	2.155	2.158
108	2.738	2.746	2.320	2.378	2.276	2.278	2.155	2.157
109	2.738	2.748	2.319	2.379	2.275	2.278	2.154	2.157
110	2.741	2.746	2.321	2.377	2.275	2.274	2.155	2.154
111	2.742	2.748	2.322	2.378	2.274	2.274	2.154	2.154
112	2.743	2.750	2.321	2.380	2.273	2.274	2.153	2.154
113	2.742	2.750	2.322	2.379	2.270	2.272	2.151	2.153
114	2.745	2.754	2.323	2.381	2.270	2.273	2.152	2.154
115	2.747	2.752	2.325	2.380	2.269	2.270	2.152	2.152
116	2.746	2.750	2.322	2.378	2.266	2.267	2.150	2.149
117	2.747	2.753	2.324	2.379	2.265	2.267	2.149	2.150
118	2.748	2.756	2.325	2.381	2.264	2.267	2.149	2.150
119	2.747	2.755	2.323	2.380	2.262	2.265	2.148	2.149
120	2.747	2.755	2.323	2.379	2.260	2.263	2.146	2.147

Table S2. Recorded reduced ion mobilities K_0 of the reactant ions $NH_4^+(H_2O)_n$, $NO^+(H_2O)_n$, $H_3O^+(H_2O)_n$ and O_2^+ in *ambient ionization mode* in air depending on reduced drift field strength E_{DR}/N at E_{RR}/N = 30 Td and E_{RR}/N = 100 Td. The reduced ion mobilities were determined from blank measurements. All other operating parameters were set according to Table 2 in the main manuscript.

	$NH_4^+(H_2O)_n$		$NO^+(H_2O)_n$		$H_3O^+(H_2O)_n$		O_2^+	
E_{RR}/N in Td	30 Td	100 Td	30 Td	100 Td	30 Td	100 Td	30 Td	100 Td
E_{DR}/N in Td	K_0 in cm²/Vs	K_0 in cm²/Vs	K_0 in cm²/Vs	K_0 in cm²/Vs	K_0 in cm²/Vs	K_0 in cm²/Vs	K_0 in cm²/Vs	K_0 in cm²/Vs
30				2.276	2.120	2.113		
31				2.281	2.123	2.115		
32				2.285	2.126	2.116		
33				2.290	2.128	2.118		
34			2.322	2.295	2.130	2.121		
35			2.309	2.301	2.133	2.124		
36			2.315	2.305	2.135	2.126		
37			2.319	2.309	2.138	2.127		
38			2.326	2.315	2.141	2.131		
39			2.330	2.321	2.143	2.135		
40			2.334	2.327	2.146	2.139		
41			2.341	2.332	2.150	2.142		
42			2.345	2.338	2.154	2.145		
43			2.350	2.345	2.157	2.150		
44			2.358	2.352	2.159	2.154		
45			2.365	2.357	2.164	2.157		
46			2.369	2.365	2.167	2.161		
47			2.374	2.373	2.171	2.166		
48			2.385	2.379	2.177	2.168		
49			2.392	2.388	2.180	2.173		
50			2.404	2.400	2.185	2.178		

51			2.413	2.408	2.188	2.180		
52			2.449	2.429	2.191	2.188		
53			2.453	2.447	2.198	2.191		
54			2.488	2.473	2.201	2.198		
55			2.492	2.492	2.207	2.201		
56			2.522	2.510	2.210	2.204		
57			2.523	2.531	2.213	2.210		
58			2.560	2.547	2.220	2.214		
59			2.573	2.567	2.224	2.222		
60			2.586	2.583	2.231	2.227		
61	2.906		2.595	2.598	2.235	2.234		
62	2.926		2.609	2.609	2.241	2.239		
63	2.947		2.620	2.622	2.247	2.245		
64	2.971		2.629	2.634	2.253	2.252		
65	2.981		2.642	2.645	2.261	2.260		
66	2.996		2.650	2.654	2.267	2.265		
67	3.008		2.658	2.663	2.274	2.272		
68	3.022		2.668	2.673	2.280	2.279		
69	3.031		2.676	2.680	2.287	2.285		
70	3.041		2.684	2.691	2.293	2.293		
71	3.050		2.691	2.698	2.299	2.300		
72	3.060		2.700	2.706	2.306	2.306		
73	3.069		2.707	2.714	2.312	2.313		
74	3.073		2.712	2.721	2.317	2.319		
75	3.082		2.721	2.728	2.324	2.325		
76	3.089		2.728	2.737	2.330	2.332		
77	3.096	3.099	2.736	2.744	2.337	2.339		
78	3.103	3.106	2.743	2.752	2.343	2.345		

79	3.109	3.113	2.750	2.760	2.349	2.352		
80	3.113	3.116	2.754	2.765	2.353	2.356		
81	3.121	3.126	2.764	2.774	2.360	2.365		
82	3.127	3.131	2.771	2.781	2.366	2.370		
83	3.132	3.136	2.777	2.786	2.371	2.375		
84	3.138	3.142	2.786	2.794	2.378	2.381		
85	3.145	3.147	2.793	2.801	2.385	2.388		
86	3.149	3.153	2.798	2.808	2.389	2.394		
87	3.154	3.160	2.805	2.816	2.395	2.401		
88	3.160	3.164	2.812	2.822	2.401	2.406		
89	3.162	3.167	2.817	2.826	2.406	2.410		
90	3.168	3.174	2.824	2.835	2.412	2.418		
91	3.174	3.178	2.832	2.840	2.419	2.424		
92	3.180	3.187	2.839	2.850	2.427	2.434		
93	3.183	3.188	2.845	2.853	2.432	2.438		
94	3.187	3.194	2.851	2.861	2.439	2.446		
95	3.192	3.198	2.858	2.866	2.447	2.453		
96	3.196	3.203	2.864	2.873	2.455	2.463		
97	3.199	3.207	2.869	2.878	2.463	2.471		
98	3.200	3.212	2.872	2.885	2.469	2.481		
99	3.206	3.212	2.880	2.887	2.481	2.488		
100	3.209	3.217	2.885	2.894	2.491	2.498		
101	3.210	3.218	2.887	2.897	2.500	2.508		
102	3.217	3.224	2.896	2.905	2.516	2.522		
103	3.219	3.227	2.900	2.909	2.528	2.533		
104	3.225	3.234	2.909	2.918	2.545	2.549		
105	3.226	3.235	2.911	2.921	2.553	2.558		
106	3.229	3.239	2.916	2.927	2.565	2.571		

107	3.234	3.242	2.923	2.933	2.579	2.583		
108	3.235	3.244	2.926	2.937	2.588	2.593	2.462	
109	3.235	3.244	2.928	2.939	2.597	2.602	2.464	
110	3.240	3.250	2.935	2.946	2.609	2.615	2.468	
111	3.242	3.250	2.939	2.948	2.619	2.623	2.471	2.470
112	3.246	3.254	2.945	2.954	2.630	2.633	2.474	2.475
113	3.248	3.256	2.950	2.959	2.639	2.643	2.476	2.479
114	3.250	3.260	2.954	2.966	2.648	2.655	2.478	2.484
115	3.253	3.264	2.959	2.971	2.656	2.664	2.480	2.487
116	3.253	3.265	2.961	2.974	2.662	2.671	2.481	2.488
117	3.257	3.265	2.967	2.976	2.672	2.676	2.484	2.489
118	3.260	3.268	2.971	2.982	2.679	2.685	2.487	2.493
119	3.262	3.270	2.976	2.986	2.686	2.693	2.489	2.495
120	3.263	3.273	2.978	2.991	2.692	2.701	2.490	2.498

Table S3. Recorded reduced ion mobilities K_0 of the ions $C_2H_3O^+$ (m/z 43), $C_3H_6OH^+$ (m/z 59), $C_3H_6O_2H^+$ (m/z 75) and $C_3H_6O_3H^+$ (m/z 91), as well as the possible TATP monomer and the adduct $TATP{\cdot}NH_4^+$ in *ammonia-doped ionization mode* in air depending on reduced drift field strength E_{DR}/N at E_{RR}/N = 30 Td and E_{RR}/N = 100 Td. The reduced ion mobilities of TATP-related product ions were determined from measurements where TATP was supplied using the gas mixing system in Figure S2 a). All other operating parameters were set according to Table 2 in the main manuscript.

	$C_2H_3O^+$ (m/z 43)		$C_3H_6OH^+$ (m/z 59)		$C_3H_6O_2H^+$ (m/z 75)		$C_3H_6O_3H^+$ (m/z 91)		Possible Monomer		$TATP{\cdot}NH_4^+$	
E_{RR}/N in Td	30 Td	100 Td	30 Td	100 Td	30 Td	100 Td	30 Td	100 Td	30 Td	100 Td	30 Td	100 Td
E_{DR}/N in Td	K_0 in cm^2/Vs	K_0 in cm^2/Vs	K_0 in cm^2/Vs	K_0 in cm^2/Vs	K_0 in cm^2/Vs	K_0 in cm^2/Vs	K_0 in cm^2/Vs	K_0 in cm^2/Vs	K_0 in cm^2/Vs	K_0 in cm^2/Vs	K_0 in cm^2/Vs	K_0 in cm^2/Vs
30							1.891		1.560		1.328	1.358
31							1.894		1.561		1.329	1.358
32							1.888		1.556		1.325	1.357
33							1.895		1.561		1.329	1.356
34				2.170			1.891		1.558		1.327	1.357
35				2.170			1.914		1.557		1.309	1.356
36				2.175			1.913		1.556		1.326	1.356
37				2.176			1.941		1.559		1.325	1.355
38				2.178			1.940		1.559		1.326	1.355
39				2.179			1.940		1.556		1.324	1.354
40				2.185			1.949		1.557		1.325	1.355
41				2.183			1.954		1.556		1.324	1.353
42				2.181			1.952		1.555		1.323	1.350
43	2.284			2.186			1.945		1.557		1.325	1.351
44	2.283			2.186			1.949		1.568		1.333	1.350
45	2.285			2.197			1.964		1.563		1.329	1.355
46	2.289			2.185			1.978		1.560		1.326	1.346
47	2.284			2.195			1.972		1.558		1.324	1.350
48	2.299			2.199			1.981		1.557		1.323	1.351

49	2.313			2.197			1.979		1.556		1.322	1.352
50	2.320			2.190			1.985		1.555		1.321	1.346
51	2.332			2.191			1.987		1.555		1.320	1.344
52	2.343			2.208			1.989		1.554		1.319	1.353
53	2.350			2.201			1.988		1.553		1.318	1.347
54	2.363			2.206			1.994		1.553		1.318	1.348
55	2.381	2.411		2.206			2.001		1.555		1.319	1.348
56	2.388	2.420		2.207	2.171		2.003		1.555		1.318	1.347
57	2.414	2.436		2.209	2.182		2.015		1.562		1.324	1.347
58	2.410	2.450		2.212	2.171		2.008		1.556		1.319	1.347
59	2.422	2.464		2.216	2.173		2.015	2.048	1.558		1.320	1.348
60	2.420	2.471		2.215	2.163		2.010	2.048	1.553		1.315	1.346
61	2.441	2.482		2.217	2.172		2.022	2.051	1.559		1.320	1.345
62	2.449	2.493		2.221	2.162		2.025	2.055	1.559		1.320	1.345
63	2.448	2.498		2.224	2.154		2.021	2.064	1.555		1.316	1.347
64	2.467	2.496		2.223	2.164		2.032	2.063	1.561		1.321	1.344
65	2.464	2.505		2.223	2.163		2.030	2.064	1.557		1.318	1.343
66	2.470	2.518		2.229	2.162		2.031	2.071	1.557		1.317	1.345
67	2.496	2.517		2.227	2.200		2.047	2.067	1.566		1.324	1.341
68	2.482	2.529		2.243	2.164	2.220	2.033	2.074	1.555		1.315	1.343
69	2.493	2.538		2.245	2.164	2.219	2.039	2.076	1.558		1.317	1.342
70	2.499	2.550		2.250	2.164	2.222	2.041	2.081	1.558		1.316	1.343
71	2.504	2.551		2.249	2.161	2.219	2.043	2.080	1.557		1.315	1.341
72	2.514	2.559		2.256	2.168	2.219	2.047	2.084	1.558		1.316	1.341
73	2.517	2.563		2.260	2.154	2.218	2.047	2.085	1.557		1.315	1.340
74	2.525	2.572		2.260	2.170	2.223	2.051	2.089	1.558		1.316	1.341
75	2.524	2.576		2.265	2.165	2.221	2.050	2.090	1.555		1.313	1.340
76	2.533	2.580		2.271	2.175	2.221	2.054	2.092	1.557		1.314	1.339

77	2.541	2.584		2.273	2.179	2.222	2.058	2.093	1.558		1.315	1.338
78	2.545	2.589		2.277	2.180	2.223	2.058	2.096	1.558		1.314	1.338
79	2.547	2.593		2.284	2.179	2.222	2.059	2.098	1.556		1.313	1.338
80	2.553	2.596		2.287	2.183	2.221	2.062	2.099	1.556		1.313	1.337
81	2.558	2.599		2.289	2.180	2.220	2.063	2.098	1.557		1.313	1.336
82	2.562	2.604		2.296	2.182	2.220	2.064	2.102	1.556		1.312	1.336
83	2.571	2.607		2.298	2.185	2.219	2.069	2.103	1.558		1.314	1.336
84	2.568	2.611		2.293	2.181	2.217	2.065	2.105	1.555		1.310	1.335
85	2.570	2.612		2.287	2.180	2.215	2.066	2.104	1.554		1.309	1.333
86	2.577	2.618		2.291	2.178	2.217	2.069	2.108	1.555		1.310	1.334
87	2.579	2.619		2.286	2.177	2.216	2.070	2.107	1.554		1.309	1.332
88	2.586	2.624		2.291	2.185	2.218	2.073	2.109	1.555		1.309	1.332
89	2.583	2.627		2.296	2.175	2.220	2.071	2.110	1.552		1.307	1.332
90	2.593	2.629		2.297	2.182	2.221	2.078	2.111	1.555		1.309	1.331
91	2.592	2.639		2.304	2.188	2.226	2.074	2.117	1.552		1.307	1.334
92	2.593	2.633		2.301	2.188	2.222	2.075	2.111	1.551		1.305	1.329
93	2.597	2.639		2.310	2.191	2.224	2.077	2.114	1.552		1.306	1.330
94	2.599	2.639	2.258	2.310	2.184	2.224	2.077	2.113	1.551		1.304	1.328
95	2.610	2.641	2.270	2.312	2.183	2.224	2.085	2.113	1.555		1.307	1.327
96	2.602	2.646	2.263	2.315	2.178	2.227	2.078	2.116	1.550		1.303	1.328
97	2.605	2.643	2.268	2.313	2.178	2.224	2.079	2.113	1.549		1.302	1.325
98	2.610	2.644	2.264	2.313	2.183	2.223	2.081	2.112	1.551		1.303	1.324
99	2.611	2.648	2.272	2.316	2.180	2.226	2.081	2.114	1.550		1.302	1.324
100	2.612	2.659	2.269	2.327	2.178	2.233	2.080	2.121	1.549		1.300	1.327
101	2.616	2.657	2.274	2.325	2.182	2.230	2.082	2.118	1.549		1.301	1.325
102	2.612	2.660	2.268	2.327	2.177	2.232	2.078	2.119	1.546		1.298	1.325
103	2.617	2.657	2.273	2.324	2.178	2.228	2.081	2.116	1.547		1.298	1.322
104	2.622	2.657	2.276	2.323	2.182	2.227	2.084	2.115	1.549		1.299	1.321

105	2.620	2.659	2.276	2.324	2.179	2.228	2.081	2.116	1.547		1.297	1.320
106	2.623	2.661	2.276	2.325	2.178	2.228	2.082	2.116	1.547		1.297	1.320
107	2.627	2.662	2.278	2.325	2.182	2.228	2.084	2.116	1.548		1.298	
108	2.626	2.663	2.280	2.325	2.180	2.227	2.083	2.116	1.546		1.296	
109	2.625	2.669	2.277	2.330	2.177	2.231	2.081	2.120	1.544		1.294	
110	2.630	2.667	2.282	2.328	2.180	2.229	2.084	2.117	1.546		1.295	
111	2.634	2.670	2.285	2.330	2.183	2.230	2.086	2.119	1.547		1.296	
112	2.629	2.675	2.280	2.334	2.176	2.232	2.081	2.121	1.544			
113	2.636	2.677	2.287	2.335	2.183	2.232	2.086	2.122	1.547			
114	2.632	2.677	2.285	2.334	2.179	2.230	2.082	2.120	1.544			
115	2.632	2.677	2.283	2.333	2.176	2.228	2.081	2.119	1.543			
116	2.632	2.681	2.283	2.335	2.178	2.229	2.080	2.120	1.542			
117	2.637	2.682	2.286	2.334	2.177	2.228	2.082	2.119	1.543			
118	2.640	2.686	2.288	2.336	2.178	2.228	2.082	2.120	1.543			
119	2.635	2.688	2.283	2.336	2.173	2.228	2.077	2.120	1.539			
120	2.641	2.688	2.287	2.335	2.176	2.225	2.080	2.118	1.541			

Table S4. Recorded reduced ion mobility K_0 of the ions $NH_4^+(H_2O)_n$ in *ammonia-doped ionization mode* in air depending on reduced drift field strength E_{DR}/N at E_{RR}/N = 30 Td and E_{RR}/N = 100 Td. The reduced ion mobilities were determined from blank measurements. All other operating parameters were set according to Table 2 in the main manuscript.

	$NH_4^+(H_2O)_n$	
E_{RR}/N in Td	30 Td	100 Td
E_{DR}/N in Td	K_0 in cm^2/Vs	K_0 in cm^2/Vs
30	2.290	2.270
31	2.295	2.275
32	2.300	2.276
33	2.306	2.284
34	2.313	2.290
35	2.320	2.299
36	2.326	2.303
37	2.332	2.311
38	2.342	2.317
39	2.347	2.329
40	2.358	2.336
41	2.367	2.349
42	2.375	2.355
43	2.383	2.367
44	2.392	2.371
45	2.401	2.389
46	2.413	2.395
47	2.424	2.412
48	2.436	2.419
49	2.451	2.433
50	2.462	2.443
51	2.478	2.454

52	2.489	2.479
53	2.507	2.493
54	2.524	2.514
55	2.547	2.531
56	2.569	2.551
57	2.595	2.581
58	2.618	2.611
59	2.653	2.638
60	2.677	2.659
61	2.707	2.685
62	2.735	2.714
63	2.767	2.742
64	2.793	2.772
65	2.823	2.796
66	2.846	2.824
67	2.869	2.849
68	2.891	2.880
69	2.916	2.892
70	2.933	2.919
71	2.949	2.939
72	2.973	2.957
73	2.988	2.975
74	3.000	2.991
75	3.016	3.024
76	3.031	3.018
77	3.042	3.035
78	3.056	3.048
79	3.066	3.065
80	3.077	3.062

81	3.083	3.089
82	3.090	3.094
83	3.103	3.106
84	3.112	3.107
85	3.120	3.120
86	3.127	3.128
87	3.132	3.131
88	3.139	3.143
89	3.146	3.144
90	3.151	3.149
91	3.156	3.162
92	3.163	3.163
93	3.165	3.176
94	3.173	3.179
95	3.179	3.180
96	3.183	3.183
97	3.188	3.188
98	3.192	3.197
99	3.197	3.206
100	3.196	3.208
101	3.209	3.213
102	3.211	3.218
103	3.215	3.216
104	3.228	3.226
105	3.224	3.230
106	3.231	3.233
107	3.233	3.240
108	3.237	3.245
109	3.248	3.246

110	3.249	3.255
111	3.239	3.253
112	3.248	3.262
113	3.247	3.259
114	3.251	3.268
115	3.254	3.263
116	3.259	3.269
117	3.259	3.275
118	3.264	3.279
119	3.262	3.281
120	3.269	3.278

S2. Setup of HiKE-IMS

The design of the HiKE-IMS is described in detail in the main manuscript. Figure S1 shows a schematic of the instrument.

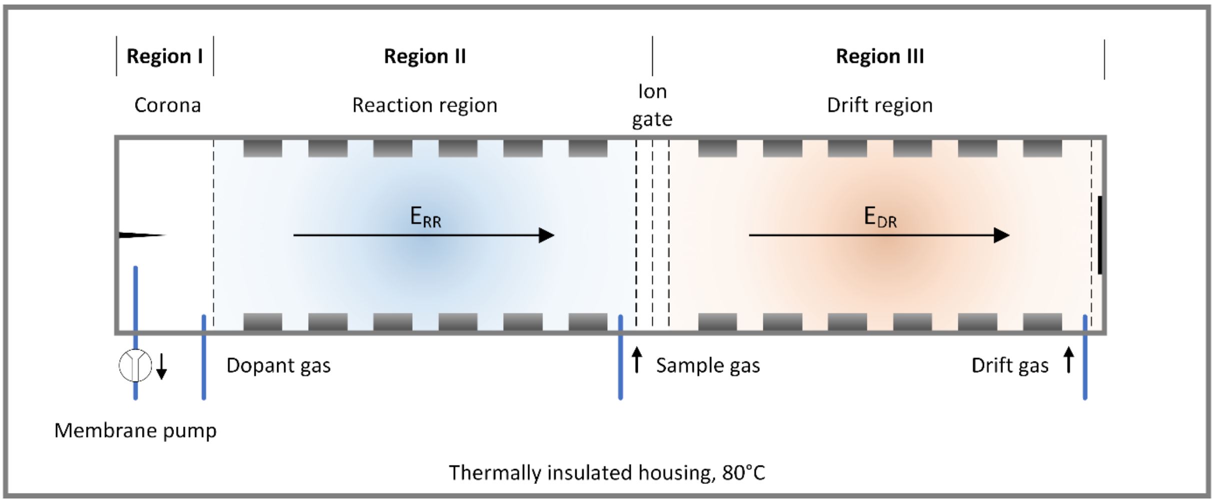

Figure S1. Schematic of the HiKE-IMS consisting of a corona discharge ion source, a reaction region, a tristate ion gate, and a drift region. The reduced electric field strengths of both the reaction region E_{RR}/N and drift region E_{DR}/N can be adjusted separately. The HiKE-IMS is located within a thermally insulated housing which is heated to 80°C. All operating parameters are given in Table 2 in the main manuscript.

S3. Gas supply

In all experiments presented in this work, TATP is placed in a sample container as shown in Figure S2. Clean nitrogen is supplied to the sample gas container with a flow rate of 250 mL/min (all gas flow rates are referenced to 293 K and 1013.25 hPa) using a mass flow controller (MFC, F-200DV-ABD, Bronkhorst Nord, Germany). For continuous sample introduction, sample gas is introduced into the HiKE-IMS through two polyetheretherketone (PEEK) capillaries connected in series. The flow rate is determined by the pressure difference between the HiKE-IMS and the sample container, as well as the capillary geometries. First, the sample passes a heated 2 m long sample capillary with an internal diameter of 1 mm, followed by a second capillary. The flow resistance and consequently the sample gas flow rate is adjusted by the second capillary with an internal diameter of 250 µm placed inside the thermally insulated housing in series with the first sample capillary to reach a sample gas flow rate of 12 mL/min. The first sample capillary is connected to a T-piece downstream of the sample container.

To evaluate the temporal system response, see Section S6, a second configuration for introducing the sample is used. Here, the sample gas can be switched between I) the gas leaving the sample gas container and II) clean nitrogen using a 3-way-valve. To maintain a constant sample gas flow rate, the pressure inside the sample container and the nitrogen line is kept constant at 20 mbar relative to ambient pressure using a pressure controller (P702CV-1K1R-RBD-33-V, Bronkhorst Nord, Germany). For this purpose, a second mass flow controller provides nitrogen with a flow rate of 250 mL/min to a T-piece connected to both the 3-way-valve and the pressure controller PC2. The excess gas flow is directed to a waste through the T-piece.

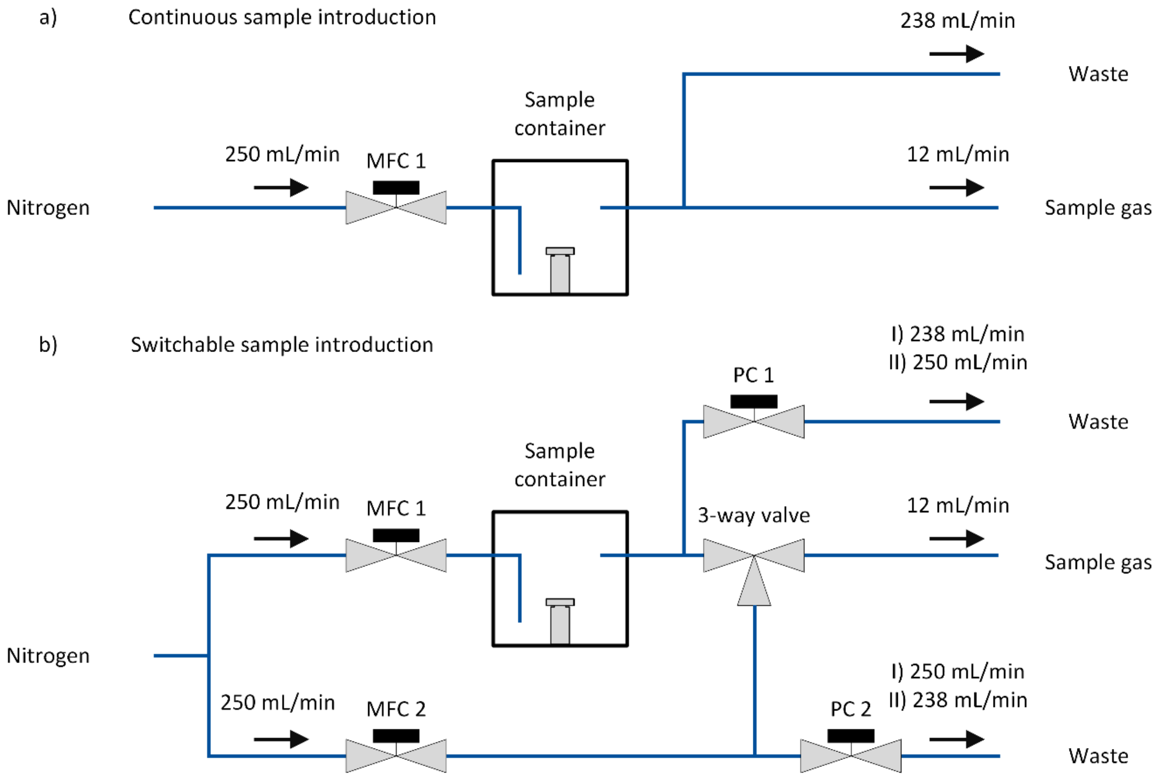

Figure S2. Schematic of the gas mixing system using mass flow controllers (MFC), pressure controllers (PC) and a 3-way valve. The TATP is placed in a sample container. Nitrogen is supplied to the sample container. a) Configuration for continuous sample introduction used in most HiKE-IMS and HiKE-IMS-MS experiments. b) Configuration for evaluating the temporal system response, see Section S6.

S4. Blank measurements

Previous publications have extensively studied the reactant ion population in *ambient ionization mode*, without any dopant gas supplied to the corona discharge ion source.[1,2] Figure S3 shows the dispersion plots of blank measurements, exhibiting only the reactant ion population in *ambient ionization mode* depending on E_{DR}/N at a) E_{RR}/N = 30 Td and b) E_{RR}/N = 100 Td. As known from previous studies, the reactant ion population consists of NH_4^+, NO^+, H_3O^+, and O_2^+.

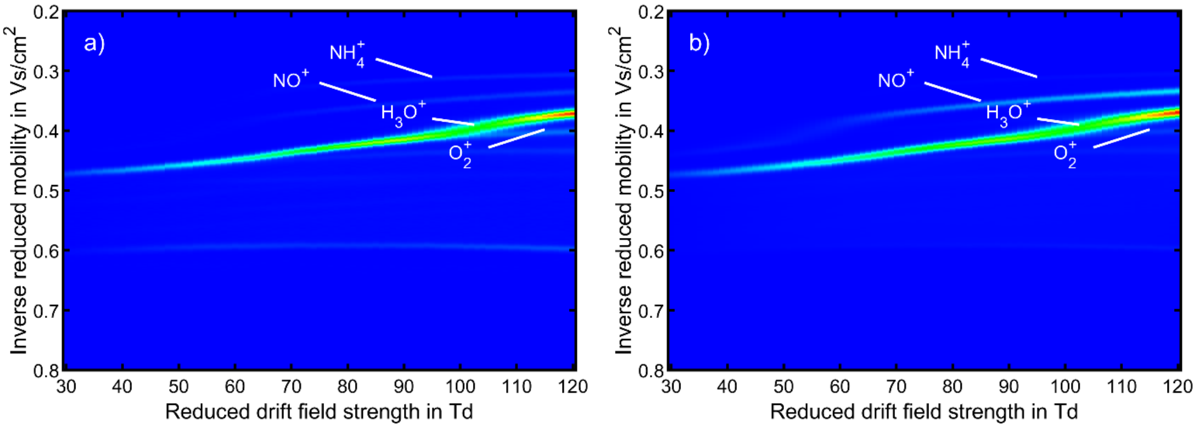

Figure S3. Blank dispersion plot recorded with the HiKE-IMS in ambient ionization mode without adding TATP at a) E_{RR}/N = 30 Td and b) E_{RR}/N = 100 Td depending on E_{DR}/N in air. The data is normalized to the maximum value of intensities of all spectra. The color denotes the intensity relative to the maximum value (red). All other operating parameters were set according to Table 2 in the main manuscript.

The addition of ammonia vapor to the corona discharge ion source when operating in *ammonia-doped ionization mode* changes the reactant ion population compared to ambient ionization mode. Figure S4 shows the dispersion plots of blank measurements providing the reactant ion population depending on E_{DR}/N at a) E_{RR}/N = 30 Td and b) E_{RR}/N = 100 Td. In *ammonia-doped ionization mode*, unlike in *ambient ionization mode*, only one dominant ion species is present at low E_{RR}/N of 30 Td, being identified as NH_4^+ by HiKE-IMS-MS. When increasing E_{RR}/N to 100 Td, three additional ion species with significant abundances can be detected. These ions are identified as the reactant ions NO^+, H_3O^+, and O_2^+.

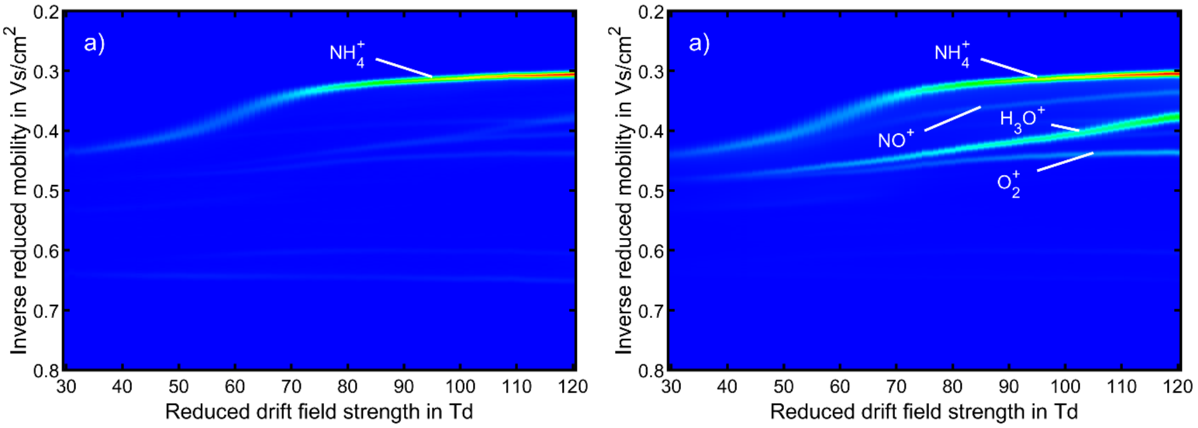

Figure S4. Blank dispersion plot recorded with the HiKE-IMS in ammonia-doped ionization mode without adding TATP at a) E_{RR}/N = 30 Td and b) E_{RR}/N = 100 Td depending on E_{DR}/N in air. The data is normalized to the maximum value of intensities of all spectra. The color denotes the intensity relative to the maximum value (red). All other operating parameters were set according to Table 2 in the main manuscript.

S5. Additional Ion Mobility Spectra

To verify whether the possible acetone-related ion species m/z 43 and m/z 59 found in HiKE-IMS in *ambient ionization mode* at $E_{DR}/N = E_{RR}/N = 100$ Td, as shown in Figure 1 of the main manuscript, correspond to the ion species found in a previous study for acetone in HiKE-IMS,[3] ion mobility spectra of acetone were recorded under the same operating conditions as for TATP in *ambient ionization mode*. For this purpose, a permeation tube filled with acetone was placed in the sample container shown in Figure S2. Figure S5 shows a comparison between a headspace measurement of acetone and TATP at $E_{DR}/N = E_{RR}/N = 100$ Td. The analysis of acetone shows two ion species with reduced ion mobilities of $K_{0,1} = 2.68$ cm^2/Vs (m/z 43) and $K_{0,2} = 2.33$ cm^2/Vs (m/z 59) in addition to the reactant ions. Thus, the same ion species as in a previous study of acetone can be found.[3] When comparing the ion mobility spectra of acetone and TATP, it is apparent that the ion species with m/z 43 and with m/z 59 have both the same mass-to-charge ratios and the same ion mobilities for both analytes. Hence, acetone and TATP possibly form the same ion species. As discussed in the main manuscript, this might either be attributed to ionization of acetone residues from synthesis in the headspace of TATP powder or to fragmentation or degradation of TATP.

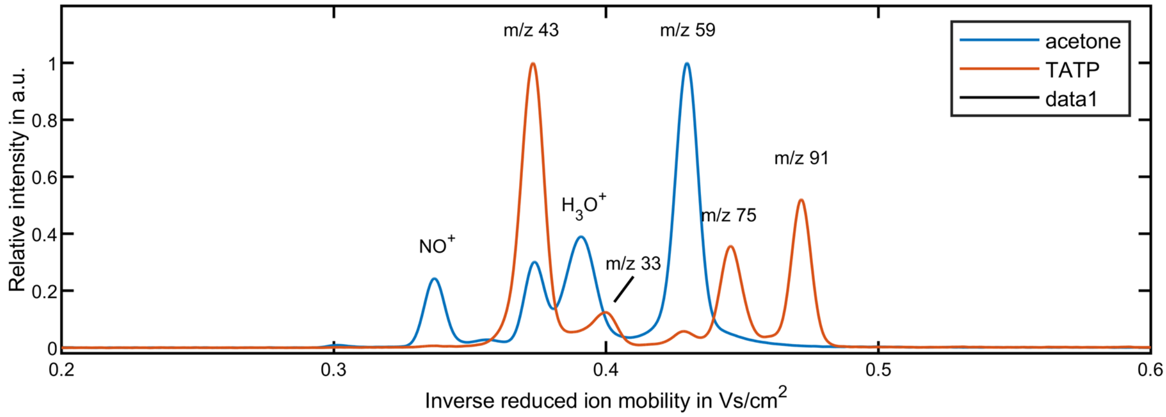

Figure S5. Comparison between ion mobility spectra in *ambient ionization mode* of headspace of both acetone and TATP at $E_{RR}/N = E_{DR}/N = 100$ Td in air obtained with standalone HiKE-IMS. All other operating parameters were set according to Table 2 in the main manuscript.

Figure S6 shows a comparison of ion mobility spectra of TATP in *ammonia-doped ionization mode* at $E_{RR}/N = 30$ Td and $E_{DR}/N = 50$ Td with $E_{DR}/N = 100$ Td. The data show that the adduct $TATP{\cdot}NH_4^+$ and the possible protonated monomer are the most abundant ion species at $E_{DR}/N = 50$ Td and form sharp peaks in the ion mobility spectrum. At higher reduced ion mobility, i.e. lower inverse reduced ion mobility, the fragments formed in the drift region form a broadened signal, since the timescale of fragmentation inside the drift region is in the same order of magnitude as the drift times under these conditions. In comparison, fragmentation appears to proceed faster at higher reduced drift field strength of $E_{DR}/N = 100$ Td and the fragments form distinct peaks in the ion mobility spectra, leaving only a slight increase in the baseline. A discussion about the identity of the fragments in *ammonia-doped ionization mode* can be found in the main manuscript.

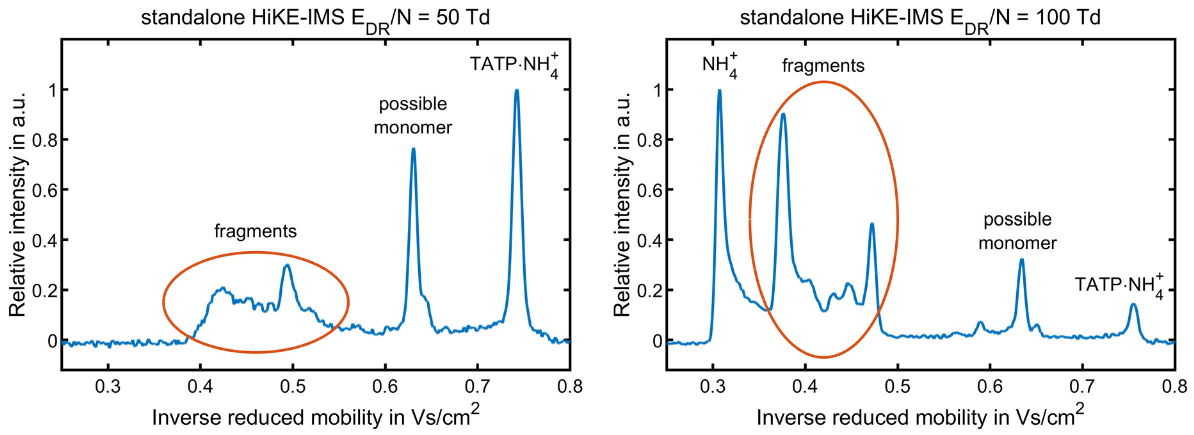

Figure S6. Comparison between positive ion mobility spectra of TATP in *ammonia-doped ionization mode* at E_{RR}/N = 30 Td and a) E_{DR}/N = 50 Td with b) E_{DR}/N = 100 Td in air. All other operating parameters were set according to Table 2 in the main manuscript.

S6. System Response Depending on Operating Temperature

To evaluate the benefit of operating HiKE-IMS at elevated temperatures, the temporal system response of HiKE-IMS to TATP operated at different temperatures of both the HiKE-IMS including the second capillary used as sample flow resistor and the first sample capillary, in the following referenced to sample inlet, are investigated. For this purpose, ion mobility spectra are recorded for a period of 180 seconds at fixed reduced electric field strengths of E_{RR}/N = 100 Td and E_{DR}/N = 100 Td. To supply the sample to the HiKE-IMS as a short sample plug, the gas mixing system is used as described in Section S3. Following a measurement time of 10 seconds where clean nitrogen is supplied to the HiKE-IMS, TATP is introduced into the HiKE-IMS for 20 seconds via the 3-way-valve in Figure S2 b). Afterwards, the valve is switched back to again provide clean nitrogen to the HiKE-IMS. In this experiment, the signal intensity of $C_3H_6O_3H^+$ (m/z 91) is analyzed over time. To eliminate any influence of temperature on signal intensity and since only the changes in signal intensity upon TATP introduction are relevant for this experiment, the signal intensities are normalized to the maximum value of each measurement.

In the first measurement shown in Figure S7, both the HiKE-IMS and the sample inlet are operated at low temperatures of 35°C each. As observed, the system's response is significantly delayed. TATP can only be detected at these low operating temperatures after 40 seconds, when the 3-way-valve has already been switched back to supply neutral nitrogen. Probably, TATP condensates in the tubes, fittings, and the instrument itself. Accordingly, the signal did not completely return to the initial value even after 180 seconds. To determine whether condensation in IMS or in the sample inlet is dominant, T_{IMS} and T_{Inlet} are individually increased. When increasing T_{Inlet} to 100°C, the signal intensity increases after 20 seconds and reaches the maximum value after 40 seconds. Therefore, the time to reach maximum signal intensity can be reduced, possibly since condensation in the first sample capillary can be mitigated. Nevertheless, the time required to purge TATP out of the HiKE-IMS remains almost unchanged as compared to operation at low temperature. When also increasing T_{IMS} from 35 °C to 60°C, the system response can be further improved, probably since the higher T_{IMS} mitigates condensation inside the HiKE-IMS and the second capillary. As expected, the system response is the shortest at maximum operating temperatures of T_{IMS} = 80°C and T_{Inlet} = 200°C. Thus, operating HiKE-IMS at high temperature compared to operation at ambient temperature significantly improves the temporal system response to TATP. Since both the temperature of the sample inlet and the temperature of the HiKE-IMS including the second capillary affect the system response, condensation needs to be considered in the entire sample line. While increasing the temperature of the sample inlet prevents sample condensation in the first sample capillary, thus decreasing the time until detecting a TATP signal, increasing the temperature of the second capillary and the HiKE-IMS helps to purge the TATP out of the instrument after exposure.

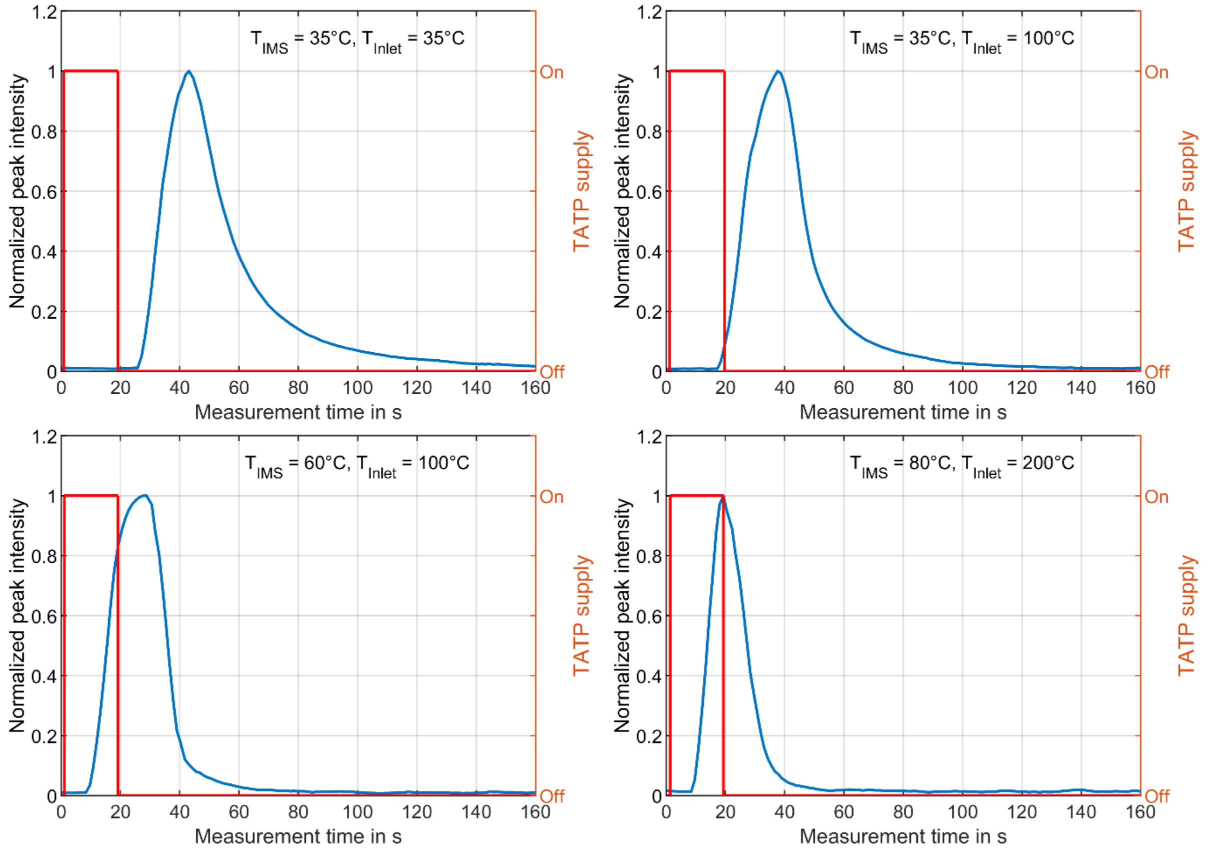

Figure S7. System response to TATP at different operating temperatures at E_{DR}/N = 100 Td and E_{RR}/N = 100 Td. a) T_{IMS} = T_{Inlet} = 35°C, b) T_{IMS} = 35°C, T_{Inlet} = 100°C, c) T_{IMS} = 60°C, T_{Inlet} = 100°C, d) T_{IMS} = 80°C, T_{Inlet} = 200°C. The plots show the intensity of the signal corresponding to $C_3H_6O_3H^+$ (m/z 91). All other operating parameters are given in Table 2 in the main manuscript.

References

(1) Allers, M., Kirk, A.T., Eckermann, M., Schaefer, C., Erdogdu, D., Wissdorf, W., Benter, T., Zimmermann, S.: Positive Reactant Ion Formation in High Kinetic Energy Ion Mobility Spectrometry (HiKE-IMS), *J. Am. Soc. Mass Spectrom.*, **31**, 1291–1301, (2020)

(2) Allers, M., Kirk, A.T., Schaefer, C., Erdogdu, D., Wissdorf, W., Benter, T., Zimmermann, S.: Field-Dependent Reduced Ion Mobilities of Positive and Negative Ions in Air and Nitrogen in High Kinetic Energy Ion Mobility Spectrometry (HiKE-IMS), *J. Am. Soc. Mass Spectrom.*, **31**, 2191-2201, (2020)

(3) Schaefer, C., Schlottmann, F., Kirk, A.T., Zimmermann, S.: Influence of Sample Gas Humidity on Product Ion Formation in High Kinetic Energy Ion Mobility Spectrometry (HiKE-IMS), *J. Am. Soc. Mass Spectrom.*, **33**, 1048–1060, (2022)
